# Macrophages Infected by a Pathogen and a Non-pathogen Spotted Fever Group *Rickettsia* Reveal Differential Reprogramming Signatures Early in Infection

**DOI:** 10.3389/fcimb.2019.00097

**Published:** 2019-04-10

**Authors:** Pedro Curto, Sean P. Riley, Isaura Simões, Juan J. Martinez

**Affiliations:** ^1^Ph.D. Programme in Experimental Biology and Biomedicine, Center for Neuroscience and Cell Biology, University of Coimbra, Coimbra, Portugal; ^2^Institute for Interdisciplinary Research, University of Coimbra, Coimbra, Portugal; ^3^CNC-Center for Neuroscience and Cell Biology, Coimbra, Portugal; ^4^Vector Borne Disease Laboratories, Department of Pathobiological Sciences, LSU School of Veterinary Medicine, Baton Rouge, LA, United States

**Keywords:** *Rickettsia conorii*, *Rickettsia montanensis*, spotted fever group *Rickettsia*, macrophages, transcriptional profiling, host-pathogen interactions

## Abstract

Despite their high degree of genomic similarity, different spotted fever group (SFG) *Rickettsia* are often associated with very different clinical presentations. For example, *Rickettsia conorii* causes Mediterranean spotted fever, a life-threatening disease for humans, whereas *Rickettsia montanensis* is associated with limited or no pathogenicity to humans. However, the molecular basis responsible for the different pathogenicity attributes are still not understood. Although killing microbes is a critical function of macrophages, the ability to survive and/or proliferate within phagocytic cells seems to be a phenotypic feature of several intracellular pathogens. We have previously shown that *R. conorii* and *R. montanensis* exhibit different intracellular fates within macrophage-like cells. By evaluating early macrophage responses upon insult with each of these rickettsial species, herein we demonstrate that infection with *R. conorii* results in a profound reprogramming of host gene expression profiles. Transcriptional programs generated upon infection with this pathogenic bacteria point toward a sophisticated ability to evade innate immune signals, by modulating the expression of several anti-inflammatory molecules. Moreover, *R. conorii* induce the expression of several pro-survival genes, which may result in the ability to prolong host cell survival, thus protecting its replicative niche. Remarkably, *R. conorii*-infection promoted a robust modulation of different transcription factors, suggesting that an early manipulation of the host gene expression machinery may be key to *R. conorii* proliferation in THP-1 macrophages. This work provides new insights into the early molecular processes hijacked by a pathogenic SFG *Rickettsia* to establish a replicative niche in macrophages, opening several avenues of research in host-rickettsiae interactions.

## Introduction

*Rickettsiae* are obligate intracellular bacteria that can cause mild to life-threatening diseases (Kelly et al., 2002). Advances in molecular techniques have allowed the detection of new and old rickettsial pathogens in new locations, suggesting an expanding distribution of reported cases and anticipating new regions of risk for rickettsioses (Richards, 2012). Spotted fever group (SFG) *Rickettsia* are recognized as important agents of human tick-borne diseases worldwide, with some members drastically differing in their ability to cause disease in humans (Uchiyama, 2012; Wood and Artsob, 2012). For example, *R. conorii* [the causative agent of Mediterranean spotted fever (MSF)] is highly pathogenic and associated with high morbidity and mortality rates, whereas *R. montanensis* has been considered as an organism with limited or no pathogenicity to humans (Walker, 1989; de Sousa et al., 2003; Galvao et al., 2005; McQuiston et al., 2012). However, the underlying mechanisms governing differences in pathogenicity by different SFG rickettsiae are still to be fully understood.

Several studies have provided evidence of non-endothelial parasitism of rickettsial species with intact bacteria being found in macrophages and neutrophils (both in tissues and blood circulation), raising the debate about the biological role of the rickettsiae-phagocyte interaction in the progression of rickettsial diseases (Walker and Gear, 1985; Walker et al., 1994, 1999; Banajee et al., 2015; Riley et al., 2016). We have recently demonstrated that the non-pathogenic *R. montanensis* and the pathogenic *R. conorii* have completely distinct intracellular fates in human THP-1 macrophages (Curto et al., 2016). *R. montanensis* are rapidly destroyed culminating in their inability to survive and proliferate in THP-1 macrophages. In contrast, *R. conorii* cells maintain the morphology of intact bacteria and establish a successful infection within these cells. Similar survival vs. death phenotypes were also observed for the virulent Breinl strain and the attenuated E strain of *R. prowazekii* in macrophage cell cultures, respectively (Gambrill and Wisseman, 1973). These results suggest that survival of rickettsial species within macrophages may be an important virulence mechanism. However, little is still known about the host and rickettsial molecular determinants responsible for these differences in growth within macrophage and its relation to pathogenesis.

Due to reductive genome evolution, *Rickettsia* are obligate intracellular pathogens, making them completely dependent on their host to survive (Sakharkar et al., 2004; Blanc et al., 2007; Darby et al., 2007). Consequently, they must have evolved different strategies to manipulate host-signaling pathways making the host environment prone to their survival and proliferation (Darby et al., 2007; Driscoll et al., 2017). Several bacterial and viral pathogens can indeed reprogram the host cell transcriptome for their benefit to survive and proliferate (Tran Van Nhieu and Arbibe, 2009; Paschos and Allday, 2010; Ashida and Sasakawa, 2014; Goodwin et al., 2015; Hannemann and Galán, 2017). However, the study of host signaling reprogramming by rickettsial species is still in its infancy.

After infection of host cells, alterations on the content of transcripts are expected as a result not only of the natural host cell response but also due to the potential manipulation of host signaling pathways by the pathogen. High-throughput transcriptomic analysis using RNA-seq has become a key tool to understand these molecular changes generated by bacterial or viral infections of eukaryotic cells (Westermann et al., 2017). In this work, we evaluate the early transcriptional alterations on THP-1 macrophages induced upon infection with the pathogenic (*R. conorii*) and the non-pathogenic (*R. montanensis*) member of SFG *Rickettsia* by RNA-seq. Since we know that *R. montanensis* is rapidly cleared by these cells (Curto et al., 2016), this experimental condition will help us to establish the typical responses of THP-1 macrophages to clear an infection vs. those induced by a member of SFG *Rickettsia* that can proliferate in these phagocytic cells. Indeed, our transcriptomic results demonstrate that a total of 470 and 86 genes were differentially altered 1 h upon infection of THP-1 macrophages with *R. conorii* and *R. montanensis*, respectively. A detailed analysis of the cellular processes affected by these genes revealed that *R. conorii* elicits changes in inflammatory responses, pro-survival pathways and transcription. Overall, these findings highlight the mechanisms that an obligate intracellular bacterial pathogen utilizes to manipulate a host cell at the transcriptional level early in the infection process, which can ultimately result in the ability of the bacterium to proliferate intracellularly within a phagocyte.

## Materials and Methods

### Cell Lines

Vero cells (CCL-81 ATCC) were grown in Dulbecco's modified Eagle's medium (DMEM, Gibco) supplemented with 10% (v/v) heat-inactivated fetal bovine serum (Atlanta Biologicals), 1 × non-essential amino acids (Corning), and 0.5 mM sodium pyruvate (Corning). THP-1 (TIB-202^TM^, ATCC) cells were grown in RPMI-1640 medium (Gibco) supplemented with 10% (v/v) heat-inactivated fetal bovine serum. Differentiation of THP-1 cells into macrophage-like cells was carried out by the addition of 100 nM of phorbol 12-myristate 13-acetate (PMA, Fisher). Cells were allowed to differentiate and adhere for 3 days prior to infection. Both cell lines were maintained in a humidified 5% CO_2_ incubator at 34°C.

### Microbe Strains

*Rickettsia conorii* isolate Malish7 and *R. montanensis* isolate M5/6 were routinely cultured in Vero cells in DMEM supplemented with 10% (v/v) heat-inactivated fetal bovine serum, 1 × non-essential amino acids, and 0.5 mM sodium pyruvate and maintained in a humidified 5% CO_2_ incubator at 34°C.

### RNA Isolation, DNase Treatment, Ribosomal RNA Depletion, and cDNA Synthesis

PMA-differentiated THP-1 cells monolayers at a cell confluency of 1.2 × 10^6^ cells per well, in 6 well plates were infected with *R. conorii, R. montanensis* at a multiplicity of infection (MOI) of 10 or maintained uninfected. Plates were centrifuged at 300 × g for 5 min at room temperature to induce contact between rickettsiae and host cells, and incubated at 34°C and 5% CO_2_ for 1 h. At the specified time point, culture medium was removed, cells were washed 1 × with PBS and total RNA was purified using SurePrep True Total RNA purification kit (ThermoFisher Scientific). DNA was removed from the RNA purification using Ambion Turbo DNase according to manufacturer's instructions. Removal of DNA contamination was verified by PCR using primers specific for the human actin gene. After DNase treatment, RNA was re-isolated using PureLink RNA Mini Kit (Ambion) according to manufacturer's instructions. RNA quality control was performed on a Fragment Analyzer (Advanced Analytical) to determine the RNA quality number (RQN). All RNA samples had a RQN > 7.2 (7.2–8.5). After confirmation of RNA structural integrity, 5 μg RNA per sample were subjected to ribosomal RNA depletion using RiboMinus^TM^ Eukaryote System v2 (Ambion) protocols. cDNA libraries were then constructed using the Ion Total RNAseq Kit v2 (Ion torrent). Sample preparation was carried out on a total of four replicates per condition.

### RNA-Seq Data Analysis

Each of the 12 samples, corresponding to four replicates per experimental condition, were independently sequenced using an Ion Proton V2 chip on Ion Chef instrument (ThermoFisher Scientific), following manufacturer's instructions. Individual read metrics per replicate per experimental condition were as follows: uninfected THP-1 cells replicates: (1) 99,693,394, (2) 83,869,431, (3) 94,336,787, (4) 97,729,124; *R. conorii*-infected THP-1 cells replicates: (1) 82,366,851, (2) 88,446,456, (3) 96,821,830, (4) 96,800,518; *R. montanensis*-infected THP-1 cells replicates: (1) 97,127,015, (2) 85,898,969, (3) 92,884,922, (4) 88,314,190. A QAQC check of the samples showed the read lengths followed a normal distribution, with average lengths between 117 and 131 bp, and an average read quality between 22 and 23. Adapters were trimmed from the samples using cutadapt (Martin, 2011), and the first 25 bp of the reads were trimmed after it was noticed several samples had spurious reads in this region. Next, STAR was used to map splice junctions to the human transcriptome, which was downloaded from ENSEMBL on 04/15/2017 (Dobin et al., 2013). The program Cufflinks and Cuffmerge was then used to map transcripts and calculate gene expression, and Cuffdiff was used to calculate which samples had genes which were statistically significantly differently expressed between conditions (Trapnell et al., 2012, 2013). Cuffdiff calculated a log_2_ fold expression for the genes in the samples using the gene expression values from Cufflinks, and a False Discovery Rate (FDR) of *p* < 0.05. In cases where the gene expression of one sample was 0, the value was set to 1 × 10^−4^ to prevent an undefined value for the log_2_ fold change calculations, and in cases where Cufflinks identified more than one isoform that mapped to reads, the first named isoform was used.

### qRT-PCR Validation

Changes to the transcriptional content of specific genes were determined by quantitative RT-PCR using SYBR Select Master Mix for CFX (Applied Biosystems). Eleven random human genes present in our RNA-seq lists were chosen to initial validate the RNAseq results and eight additional genes were selected for further validation of *R. conorii*-induced responses. Primers that generate PCR products smaller than 90 nucleotides were designed for each specific gene. PCR reactions with the respective primer set and using human genomic DNA isolated from THP-1 cells as template were carried out as follows: 95°C for 2 min, followed by 35 cycles of 95°C for 15 s, 58°C for 15 s and 72°C for 60 s. Amplified PCR products were cloned into pCR2.1 using TOPO TA cloning kit (Invitrogen) according to manufacturer's instructions and confirmed by DNA sequencing. Plasmids were used to generate standard curves for each specific gene of interest (GOI). The quantity of transcript for each gene present in each cDNA library was determined by qPCR using the following conditions: 50°C for 2 min, 95°C for 2 min, followed by 40 cycles of 95°C for 15 s, 58°C for 15 s, and 72°C for 60 s followed by melting curve using a LightCycler 480 II (Roche). qRT-PCR-derived fold change values are expressed as in Equation 1:

$$\begin{array}{l} fold\;change=LOG2\;\left(Exp.cond.2/Exp.\;cond.1\right)\\ \;=\left[\frac{\left(GOI\;Exp.\;cond.\;2/Reference\;gene\;Exp.\;cond.\;2\right)}{\left(GOI\;Exp.\;cond.\;1/Reference\;gene\;Exp.\;cond.\;1\right)}\right] \end{array}$$

Glucose-6-phosphate dehydrogenase (G6PD) and beta-2-microglobulin (B2M) were used as reference genes to normalize the results between the different experimental conditions (Eisenberg and Levanon, 2013; Leisching et al., 2016).

### Bioinformatics Analysis

Principal Component Analysis (PCA) was performed by importing the mapped read (BAM) files into a server running Partek® Flow® Software, version 6.0.17, Copyright 2017. The Volcano Plots were created from the output from CuffDiff, with custom python, and R scripts. Significantly differentially expressed genes were uploaded into DAVID Bioinformatics Resources 6.8 (https://david.ncifcrf.gov/home.jsp) to categorize genes according to biological function, host cellular pathways and cellular component gene ontology (GO) terms (Huang da et al., 2009). Significantly differentially expressed genes were also uploaded into Ingenuity Pathway Analysis (IPA) (QIAGEN Inc., https://www.qiagenbioinformatics.com/products/ingenuity-pathway-analysis) to identify significant altered canonical pathways or downstream disease/functions (Krämer et al., 2014). Activation or inhibition of predicted canonical pathways and disease/function were determined by *Z*-scores calculated by IPA. Positive *Z*-scores (>2.0) predict activation whereas negative *Z*- scores (< −2.0) predict inhibition. Functional protein association networks were evaluated using STRING 10.5 (http://string-db.org/) with high confidence (0.7) parameters (Szklarczyk et al., 2017).

### TNFα Activation of THP-1 Cells

PMA-differentiated THP-1 cells at 5 × 10^4^ THP-1 cells per well were infected with *R. conorii* or *R. montanensis* (MOI = 10), and centrifuged at 300 × g for 5 min at room temperature to induce contact. 24 h after infection, 5 μg/mL *Escherichia coli* O26:B6 Lipopolysaccharide (Invitrogen) in culture media or media alone was added, and incubated for 24 additional hours. 48 total hours after infection, the media was removed, and TNFα concentration was determined by ELISA with Maxisorp plates (Nunc), Human TNFα Duo Set (R&D Systems), and OptiEIA TMB substrate (BD biosciences). Absorbance was measured at 450 mn and standard curve generated with recombinant human TNFα (R&D Systems).

### PARP-1 Cleavage Assay

PMA-differentiated THP-1 cells were seeded on glass coverslips in 24-well plates at 2 × 10^5^ cells per well. THP-1 monolayers were then infected with *R. conorii* (MOI = 2.5), the plates were centrifuged at 300 × g for 5 min at room temperature to induce contact, and subsequently incubated for 1, 3, and 5 days at 34°C and 5% CO_2_. As a control, uninfected THP-1 macrophages were always kept at the same experimental conditions. When mentioned, *R. conorii*-infected and uninfected cells were incubated with staurosporine (EMD Biosciences) at a final concentration of 750 nM to induce intrinsic apoptosis during 4 h. At each specific time point, *R. conorii*-infected and uninfected THP-1 macrophages were washed 1 × with 1 mL of PBS, and fixed in 4% PFA for 20 min prior to staining. After permeabilization with 0.1% Triton X-100 and blocking with 2% BSA, cells were then incubated with rabbit anti-cleaved poly(ADP-ribose) polymerase (PARP) (1:400) (Cell Signaling Technology) and mouse anti-*R. conorii* 5C7.31 (1:1,500) antibodies for 1 h, washed 3 × in PBS, and then incubated in PBS containing 2% BSA, Alexa Fluor 488-conjugated goat anti-rabbit IgG (1:1,000) (ThermoFisher Scientific), Alexa Fluor 594-conjugated goat anti-mouse (1:1,000) (ThermoFisher Scientific), and DAPI (1:1,000) (ThermoFisher Scientific). After washing 3 × with PBS, glass coverslips were mounted in Mowiol mounting medium and preparations were viewed on a LEICA DM 4000 B microscope equipped with Nuance FX multispectral imaging system using a final X40 optical zoom and processed with Image J software (https://imagej.nih.gov.ij/).

### Statistical Analysis

**Figure 1:**
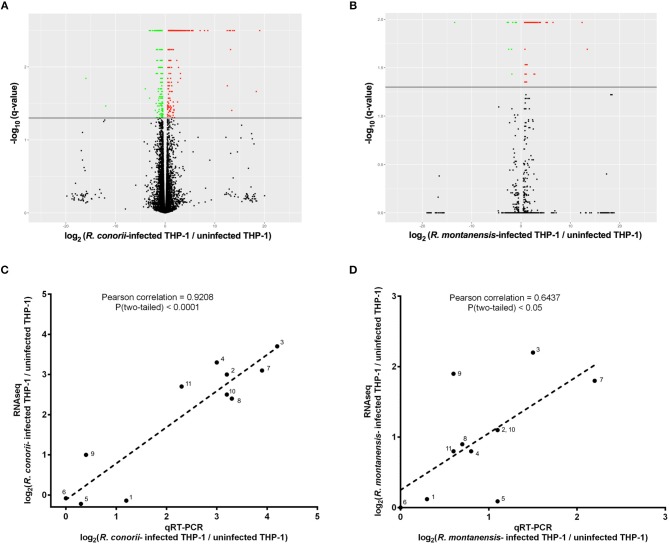
SFG *Rickettsia* trigger reprogramming in THP-1 macrophages early in infection. **(A,B)** Volcano plots of log_2_ fold change ratio of the expression levels in *R. conorii*- **(A)** and *R. montanensis*- **(B)** infected THP-1 macrophages over that in uninfected cells plotted against the -log_10_ (*q*-value). Statistically differentially expressed genes found in increased abundance and in decreased abundance are represented in red and green, respectively (FDR < 0.05). See also Tables S1, S2. **(C,D)** Validation of RNA-seq data by comparing the transcriptional fold changes of 11 randomly selected genes determined by RNA-seq and an independent method (q-RT-PCR) for *R. conorii*- **(C)** and *R. montanensis*- **(D)** infected cells. Pearson analysis of correlation and respective significant test (two-tailed) were performed in GraphPad Prism. *R. conorii*-infected: *r* = 0.9208, *N* = 11, *p* < 0.0001 and *R. montanensis*-infected: *r* = 0.6437, *N* = 11, *p* < 0.05. Gene labeling: 1-B2M; 2-BTG2; 3-CD69; 4-EGR1; 5-EMC7; 6-G6PD; 7-IER3; 8-KLF10; 9-MTRNR2L6; 10-OTUD1; 11-PP1R15A. See also Table S3.

Correlation between qRT-PCR and RNAseq results was performed by Pearson analysis of correlation in GraphPad Prism (GraphPad Software, Inc) together with a significant test (two-tailed test). Pearson correlation coefficients and respective *P* values can be found in Figures 1C,D.

TNFα activation assays experiments were performed twice with each individual experiment done in triplicate for each experimental condition. Statistical analysis was performed by Mann Whitney test using GraphPad Prism (GraphPad Software, Inc). Results are shown as mean ± SD and differences were considered non-significant (ns) at *P* > 0.05 or significant at ^*^P ≤ 0.05, ^**^P ≤ 0.01, ^***^P ≤ 0.001.

PARP-cleavage experiments were done in quadruplicate and at least 100 mammalian nuclei were counted for each independent experiment. Results of each experiment were expressed as the percentage of cleaved PARP-positive cells. Statistical analysis was performed by Mann Whitney test using GraphPad Prism (GraphPad Software, Inc). Results are shown as mean ± SD and differences were considered non-significant (ns) at *P* > 0.05 or significant at ^*^P < 0.05.

## Results

### SFG *Rickettsia* Trigger Macrophage Reprogramming Early in Infection

The drastic intracellular phenotypic differences between *R. montanensis* and *R. conorii* in THP-1 macrophages as early as 1 h post-infection (Curto et al., 2016) suggest early transcriptomic alterations, either as a result of host cell response to infection or bacterial manipulation. To further elucidate the host responses that contribute to *R. conorii*-specific patterns of intracellular proliferation, we performed a global profiling of early transcriptional responses of cultured human THP-1 macrophages challenged with *R. conorii* and *R. montanensis* (MOI = 10). Supported by our previous findings that *R. montanensis* are rapidly destroyed in these cells (Curto et al., 2016), the inclusion of this experimental condition permits to establish what the normal macrophage responses to an avirulent *Rickettsia* species are vs. those induced by the pathogen *R. conorii*. RNA harvested at 60 min post-infection was subjected to whole genome transcriptomic analysis and compared to uninfected cells, processed in parallel. Read metrics indicated high-quality results for RNAseq, with averages of 93,907,184 (uninfected cells), 91,108,913 (*R. conorii*-challenged), and 91,056,274 (*R. montanensis*-challenged) reads in the four replicates for each experimental condition. Principal component analysis (PCA) was carried out to assess the sample correlations using the expression data of all genes (Figure S1). Cuffdiff was then used to determine significantly differentially expressed (DE) genes between infected and uninfected conditions with a *p*-value cutoff of < 0.05 (FDR *p* < 0.05), and results were shown as log_2_ fold change. In total, 470 genes were filtered to be expressed at significantly higher levels (*n* = 267) or lower levels (*n* = 203) in *R. conorii*-infected macrophages (Figure 1A; Table S1). On the other hand, 86 genes were filtered to be expressed at significantly higher levels (*n* = 75) or lower levels (*n* = 11) in *R. montanensis*-infected cells (Figure 1B; Table S2). These results indicate that infection with either the pathogenic (*R. conorii*) or the non-pathogenic (*R. montanensis*) SFG *Rickettsia* result in transcriptomic changes in the host as early as 1 h post-infection. To validate the RNA-seq results, 11 human genes were chosen for individual analysis by an independent experimental method. The genes were randomly selected to represent transcripts present in both infection conditions. The amount of transcript for each gene was determined for all experimental conditions and log_2_ q-RT-PCR-derived fold change for each situation was determined according to equation 1 (Methods). Log_2_ fold change values are shown in Table S3. The q-PCR data for the analyzed genes were then compared to the fold change values obtained by RNA-seq, and the results from each quantification method demonstrate a very strong [*r* > 0.8, *P* value (two-tailed test) < 0.0001] and a strong correlation [*r* > 0.6, *P* value (two-tailed test) < 0.05] between experimental methods (Figures 1C,D) for *R. conorii*- and *R. montanensis*-infected THP-1 macrophages, respectively, thereby validating the transcriptional changes obtained by RNA-seq.

### *Rickettsia conorii* Infection Promotes a Robust Modulation of Host Gene Expression Profiles

**Figure 2:**
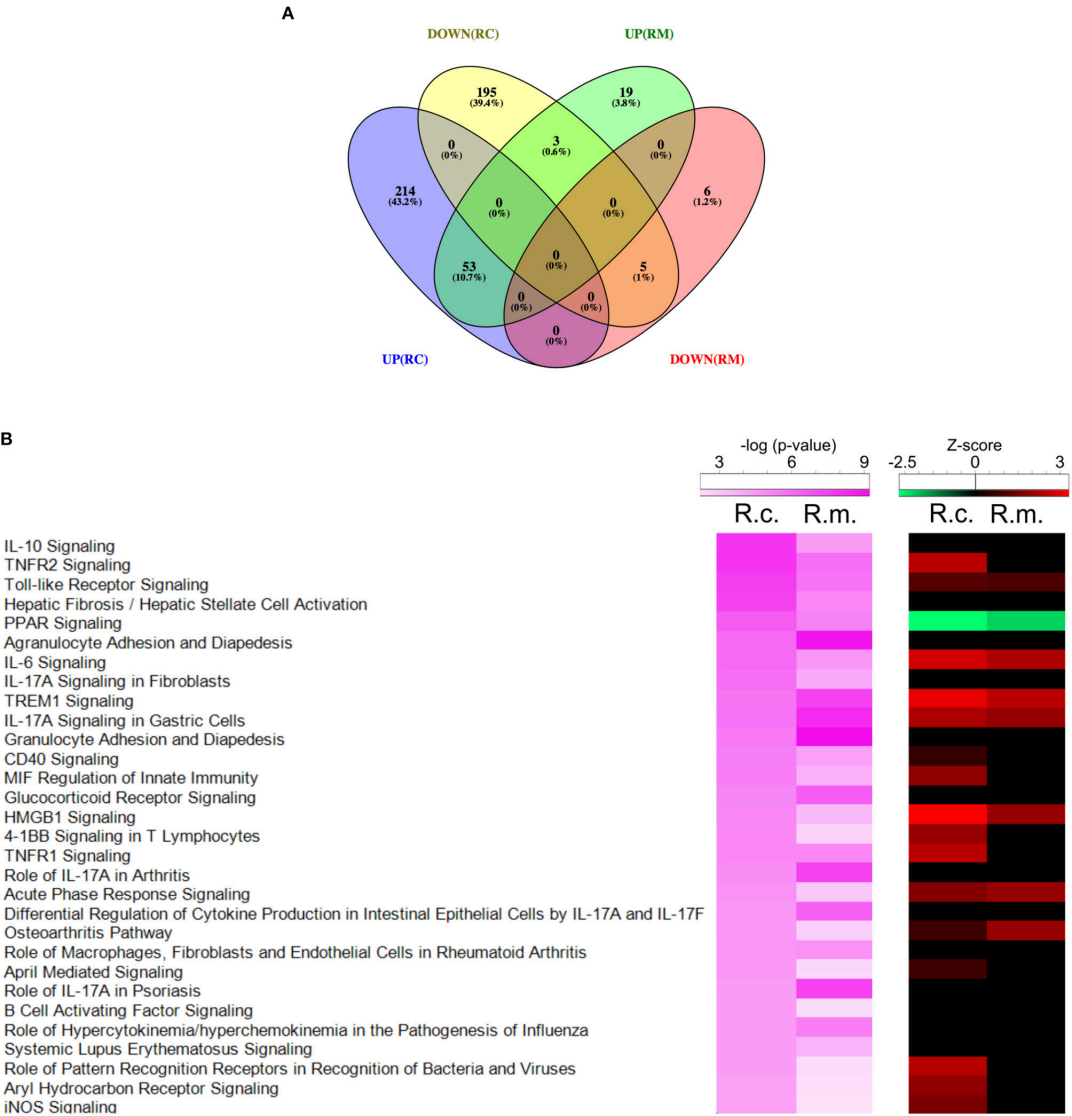
Gene expression patterns stimulated by infection of THP-1 macrophages with *R. conorii* or *R. montanensis* reveal a more robust modulation by the pathogenic species. **(A)** Venn diagram depicting the number and distribution of specific and common DE genes in each experimental condition. UP means increased abundance, DOWN means decreased abundance, RC is *R. conorii*-infected cells and RM is *R. montanensis*-infected cells. See also Table S4. **(B)** Prediction of the activation/inhibition state of the top 30 canonical pathways in *R. conorii*-infected cells (R.c.) ranked by -log(*p*-value) and corresponding prediction states in *R. montanensis*-infected cells (R.m.) according to Ingenuity Pathway Analysis (IPA). Pink colored heatmap shows the top 30 canonical pathways for *R. conorii*-infected cells (R.c.) (ranked by -log(*p*-value) and the respective -log(*p*-value) for the correspondent pathway in *R. montanensis*-infected cells (R.m.). Red-green heatmap shows the prediction of activation (red)/inhibition (green) state (*Z*-score) in *R. conorii*-infected cells (R.c.) and *R. montanensis*-infected cells (R.m.). Pathways are considered to be inhibited or activated for *Z*-scores values < −2.0 or > 2.0, respectively. See also Table S4.

The challenge of THP-1 macrophages with *R. conorii* and *R. montanensis* resulted in a total of 495 host genes of which the transcript levels were considered statistically DE at 1-h post infection when compared to uninfected cells. After sorting out this differential gene expression per experimental condition, four different groups of genes were identified (Figure 2A; Table S4): 409 genes showed changes in abundance only in the *R. conorii* dataset (214 with increased abundance and 195 with decreased abundance) and were designated as *R. conorii*-specific; 61 genes were common to infection by both rickettsial species (53 with increased abundance and 5 with decreased abundance), with 3 genes in this group showing differential abundance between datasets; and 25 genes (19 with increased abundance and 6 with decreased abundance) were identified as *R. montanensis*-specific.

Gene ontology (GO) analysis of the DE genes was carried out using DAVID Bioinformatics Resources (Huang da et al., 2009). Genes were categorized according to biological function, canonical pathways, and cellular component. The top GO terms enriched in each category listed by *p*-value are shown in Tables 1, 2. Analysis of the 58 DE genes commonly altered by the infection of either *R. conorii* or *R. montanensis* (Table 1) revealed the enrichment of genes involved in inflammatory response, cellular response to tumor necrosis factor (TNF), cellular response to lipopolysaccharide, immune response, among others. DE genes were also categorized into several canonical pathways, which include TNF signaling, toll-like receptor (TLR) signaling pathway, Nuclear Factor-κB (NF-κB), chemokine signaling, and cytokine-cytokine receptor interaction. Moreover, a higher representation of transcripts corresponding to extracellular proteins was observed. The three genes that are inversely DE between *R. conorii* and *R. montanensis*-infected cells correspond to non-coding RNAs (RNU1-148P; RNU5A-1; RNU5D-1).

**Table 1:** Categorization of the 58 DE genes commonly altered by the infection of either *R. conorii* or *R. montanensis*.

	**Number of genes**	**% Gene count**	***p*-value**
**COMMON DE GENES (58 GENES)**			
**GO biological process**			
Inflammatory response (GO:0006954)	13	22.4	1.1 × 10^−11^
Neutrophil chemotaxis (GO:0030593)	8	13.8	8.0 × 10^−11^
Cellular response to tumor necrosis factor (GO:0071356)	8	13.8	3.0 × 10^−9^
Chemokine-mediated signaling pathway (GO:0070098)	7	12.1	8.1 × 10^−9^
Cellular response to interleukin-1 (GO:0071347)	7	12.1	8.1 × 10^−9^
Immune response (GO:0006955)	10	17.2	1.9 × 10^−7^
Positive regulation of ERK1 and ERK2 cascade (GO:0070374)	7	12.1	1.8 × 10^−6^
Cellular response to lipopolysaccharide (GO:0071222)	6	10.3	4.0 × 10^−6^
Negative regulation of cell proliferation (GO:0008285)	8	13.8	1.8 × 10^−5^
Negative regulation of transcription from RNA polymerase II (GO:0000122)	9	15.5	1.1 × 10^−4^
G-protein coupled receptor signaling pathway (GO:0007186)	9	15.5	5.2 × 10^−4^
Positive regulation of transcription from RNA polymerase II promoter (GO:0045944)	9	15.5	9.2 × 10^−4^
Signal transduction (GO:0007165)	8	13.8	1.1 x 10^−2^
**GO cellular component**			
Extracellular region (GO:0005576)	15	25.9	3.0 × 10^−6^
Extracellular space (GO:0005615)	12	20.7	8.4 × 10^−5^
**KEGG canonical pathways**			
TNF signaling pathway (hsa04668)	11	19.0	2.0 × 10^−12^
Salmonella infection (hsa05132)	8	13.8	1.5 × 10^−8^
Toll-like receptor signaling pathway (hsa04620)	8	13.8	8.5 × 10^−8^
NF-kappa B signaling pathway (hsa04064)	7	12.1	6.3 × 10^−7^
Rheumatoid arthritis (hsa05323)	7	12.1	6.7 × 10^−7^
Legionellosis (hsa05134)	6	10.3	1.4 × 10^−6^
Chagas diseases (hsa05142)	7	12.1	1.8 × 10^−6^
Chemokine signaling pathway (hsa04062)	8	13.8	3.9 × 10^−6^
Cytokine-cytokine receptor interaction (hsa04060)	8	13.8	1.6 × 10^−5^
HTLV-I infection (hsa05166)	6	10.3	2.3 x 10^−3^

*Common DE genes are categorized using DAVID Bioinformatic Resources 6.8 according to GO terms: biological process and cellular component, and KEGG canonical pathways*.

**Table 2:** Categorization of the 409 DE genes uniquely altered in *R. conorii*-infected THP-1 macrophages.

	**Number of genes**	**% Gene count**	***p*-value**
***R. conorii*****-SPECIFIC DE GENES (409 GENES)**			
**GO biological process**			
Positive regulation of transcription from RNA polymerase II promoter (GO:0045944)	35	8.6	1.2 × 10^−6^
Inflammatory response (GO:0006954)	20	4.9	2.0 × 10^−6^
Negative regulation of transcription from RNA polymerase II promoter (GO:0000122)	25	6.1	9.0 × 10^−5^
Response to lipopolysaccharide (GO:0032496)	11	2.7	9.3 × 10^−5^
Positive regulation of cell migration (GO:0030335)	11	2.7	1.2 × 10^−4^
Angiogenesis (GO:0001525)	12	2.9	3.4 × 10^−4^
Positive regulation of gene expression (GO:0010628)	13	3.2	3.7 × 10^−4^
Transcription from RNA polymerase II promoter (GO:0006366)	19	4.7	3.9 × 10^−4^
Cell adhesion (GO:0007155)	17	4.2	8.9 × 10^−4^
Negative regulation of apoptotic process (GO:0043066)	16	3.9	2.2 × 10^−3^
Apoptotic process (GO:0006915)	18	4.4	3.0 × 10^−3^
Positive regulation of transcription, DNA-templated (GO:0045893)	16	3.9	6.8 × 10^−3^
Immune response (GO:0006955)	13	3.2	1.7 × 10^−2^
Negative regulation of cell proliferation (GO:0008285)	12	2.9	2.6 × 10^−2^
Cell proliferation (GO:0008283)	11	2.7	3.6 × 10^−2^
Innate immune response (GO:0045087)	11	2.7	8.7 × 10^−2^
**GO cellular component**			
Integral component of plasma membrane (GO:0005887)	34	8.3	1.7 × 10^−3^
Extracellular space (GO:0005615)	31	7.6	3.2 × 10^−3^
Extracellular exosome (GO:0070062)	53	13.0	1.2 × 10^−2^
Extracellular region (GO:0005576)	32	7.8	3.1 × 10^−2^
Nucleus (GO:0005634)	88	21.5	3.8 × 10^−2^
**KEGG Canonical pathways**			
Transcriptional misregulation in cancer (hsa05202)	12	2.9	5.2 × 10^−5^
Pertussis (hsa05133)	6	1.5	5.9 × 10^−3^
MAPK signaling pathway (hsa04010)	11	2.7	5.9 × 10^−3^
Osteoclast differentiation (hsa04380)	8	2.0	4.1 × 10^−3^
HTLV-I infection (hsa05166)	9	2.2	4.4 × 10^−2^
TNF signaling pathway (hsa04668)	6	1.5	2.4 × 10^−2^
NF-kappa B signaling pathway (hsa04064)	7	1.7	2.2 × 10^−2^
FoxO signaling pathway (hsa04068)	7	1.7	1.7 × 10^−2^
Viral carcinogenesis (hsa05203)	9	2.2	1.4 × 10^−2^
microRNAs in cancer (hsa05206)	11	2.7	1.3 × 10^−2^

*R. conorii-specific DE genes are categorized using DAVID Bioinformatic Resources 6.8 according to GO terms: biological process and cellular component, and KEGG canonical pathways*.

On the other hand, analysis of the 409 DE genes designated as *R. conorii*-specific (Table 2) revealed a differential abundance of genes involved in both positive and negative regulation of transcription from RNA polymerase II promoter, inflammatory response, response to lipopolysaccharide, positive regulation of gene expression, and regulation of apoptotic process. Moreover, *R. conorii*-specific genes map to several canonical pathways, such as mitogen-activated protein kinase (MAPK), TNF, and NF-κB signaling. Overrepresentation of genes categorized to nuclear localization is also observed. For the 25 DE genes designated as *R. montanensis-*specific, no significant enrichment was detected with very few genes categorized according to DAVID databases (Table S5).

To gain more insight about the datasets, significantly DE genes were also uploaded into IPA, which combines differential gene expression data with the Ingenuity Pathway Knowledge Base to determine altered canonical pathways, upstream regulators and predicted downstream disease/functions (Krämer et al., 2014). The list of altered canonical pathways and their predicted activation/inhibition scores for *R. conorii*- and *R. montanensis*-infected THP-1 macrophages can be found in Table S4. To better understand similarities and differences on the activation/inhibition state of signaling pathways, the top 30 canonical pathways in *R. conorii*-infected cells (aligned by *p*-value) were compared to the corresponding *p*-values observed for the same pathways in *R. montanensis*-infected cells (pink-colored heatmaps). The predicted activation/inhibition scores (Z-score) were also listed (red-green colored heatmaps) (Figure 2B). As illustrated in the heatmaps, the pattern of altered canonical pathways differed between experimental conditions. The activation of signaling pathways such as High Mobility Group Protein B1 (HMGB1), Triggering Receptor Expressed on Myeloid cells-1 (TREM1), interleukin 6 (IL-6), and acute phase response signaling was predicted in both *R. conorii*- and *R. montanensis*-infected macrophage-like cells, which is consistent with an augmented inflammatory response upon infection. However, several other pathways such as Tumor Necrosis Factor Receptor 1 (TNFR1) and Tumor Necrosis Factor Receptor 2 (TNFR2) signaling, and role of pattern recognition receptors in recognition of bacteria and viruses were predicted to be activated only in *R. conorii*-infected cells.

### *Rickettsia conorii* Infection Switches Immune Signals in Macrophage-Like Cells Into a Hyporesponsive State

**Figure 3:**
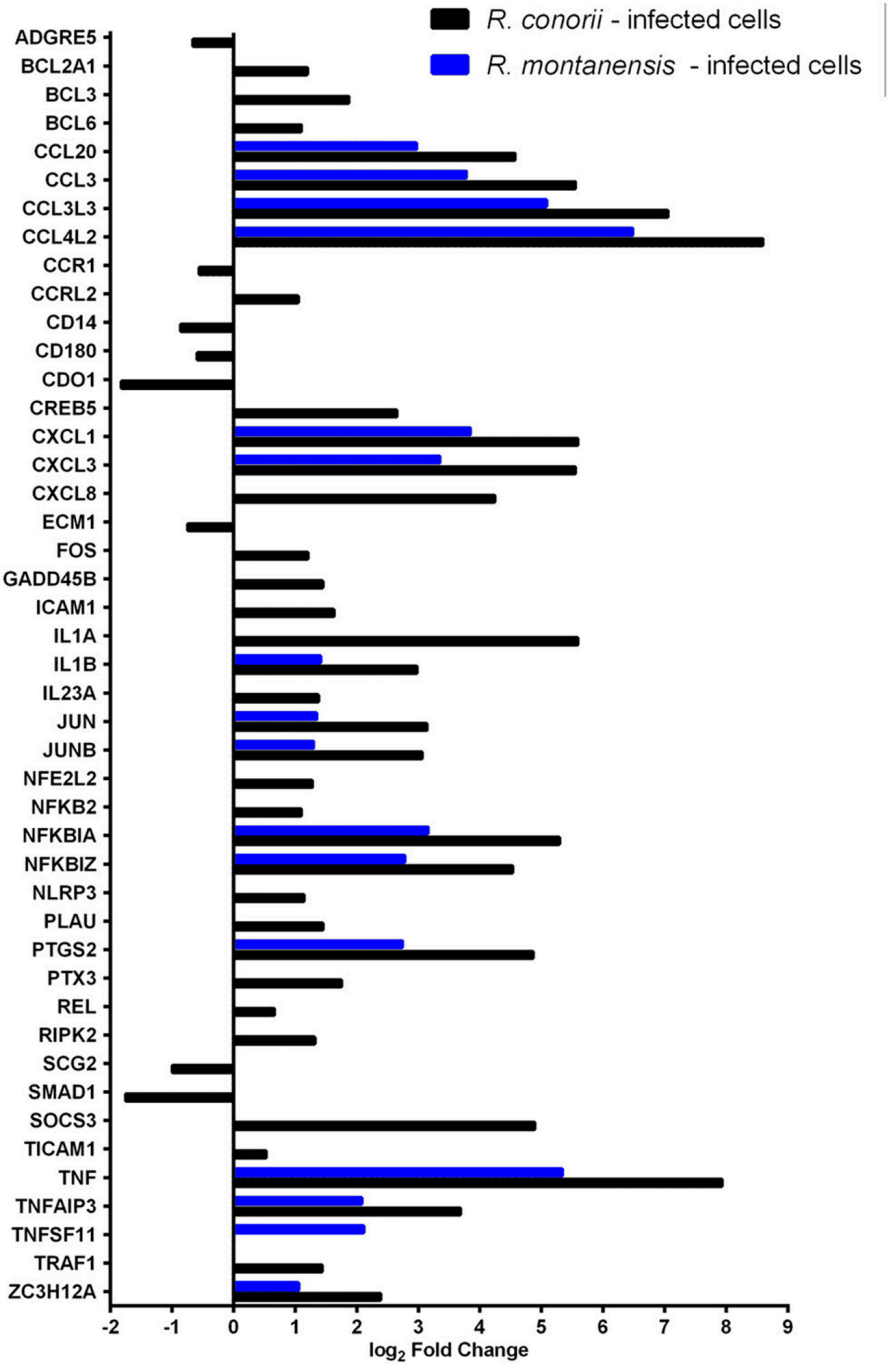
*Rickettsia conorii* and *R. montanensis* differentially modulate innate immune responses during THP-1 macrophage infection. Combined list of the individual DE genes (and respective log_2_ fold change values) categorized with the GO term inflammatory response (GO:0006954) and KEGG pathways: TLR signaling pathway (hsa04620), NF-κB signaling pathway (hsa04064), and TNF signaling pathway (hsa04668). DE genes in THP-1 macrophages infected with *R. conorii* are shown in black bars and in *R. montanensis* are shown in blue. The absence of bar means that the fold change of that gene for the respective experimental condition was not considered statistically significant. See also Table S6.

Central to the modulation of inflammatory and immune responses are TLR, NF-κB, and TNF signaling pathways (Karin and Lin, 2002; Li and Verma, 2002; Kawai and Akira, 2010; Brenner et al., 2015; Kalliolias and Ivashkiv, 2016). We observed differential expression of several genes grouped to inflammatory responses (GO:0006954), TLR (hsa04620), NF-κB (hsa04064) and TNF (hsa04668) signaling in THP-1 cells infected with *R. conorii and R. montanensis* (Figure 3; Table S6). A subset of these genes was further validated by qRT-PCR for *R. conorii*-infected cells demonstrating a strong correlation between experimental methods (*r* = 0.7786, *N* = 8, *P* value (two-tailed test) < 0.05) (Figure S2; Table S7). DE genes with increased abundance in transcripts in both *R. conorii* and *R. montanensis* datasets (although at different levels) include the pro-inflammatory cytokines TNFα and IL1β, as well as the chemokines CCL20, CCL3L3, CCL3, CCL4L2, CXCL1, CXCL3, and CXCL8 that can shape the recruitment of immune cells to the site of infection (Newton and Dixit, 2012). Other genes implicated in the modulation of NF-κB signaling (TNFAIP3 and NFKBZ) or involved in the synthesis of inflammatory mediators (PTGS2), as well as genes involved in both negative (NFKBIA and TNFAIP3) and positive (TNF and IL1B) regulatory loops of the NF-κB pathway, were also induced by both rickettsial species.

Importantly, several genes were found to be DE only in *R. conorii*-infected cells. This group includes the upregulated cytokine IL1α, the subunit IL23a, and TLR adaptor molecule 1 (TICAM1, also known as TRIF). TICAM1 is critical for TLR3- and TLR4-mediated signaling pathways that can lead to the activation of late-phase NF-κB and following induction of inflammatory cytokines (Yamamoto et al., 2003; Kawai and Akira, 2010). Also, eight genes were found in decreased abundance in the *R. conorii* dataset only (ADGRE5, CCR1, CD14, CD180, CDO1, ECM2, SCG2, SMAD1). ADGRE5 is considered a critical mediator of host defense, playing essential roles in leukocyte recruitment, activation, and migration (Gray et al., 1996; Leemans et al., 2004). CCR1 blocking impairs host defenses by perturbing the cytokine response during Herpes simplex type 2 infection (Sørensen and Paludan, 2004). Transcripts encoding proteins that have been reported as involved in host cell survival, such as GADD45B, TRAF1, BCL2A1, CXCL8, and PLAU were also found in increased abundance only in the *R. conorii-*infected dataset.

TNF signaling cascades are initiated with the binding of soluble TNF to either of its receptors (TNFR1 or TNFR2). However, signaling cascades generated by each receptor are markedly different (Brenner et al., 2015). IPA revealed that both TNFR1 and TNFR2 signaling pathways are predicted to be activated only in *R. conorii*-infected THP-1 macrophages (*Z*-scores of 2.449; *p*-value of 9.77 × 10^−6^ and 1.15 × 10^−8^, respectively) (Table S4), anticipating significant differences in host signaling responses through both pathways, between bacterial species. It has been reported that TNFR1 signaling can result in either cell survival or cell death depending on downstream signaling events and cellular context, and TNFR2 signaling promotes cell survival (Lee and Choi, 2007; Brenner et al., 2015; Wan et al., 2016). Infection with *R. conorii* resulted in an increased abundance of mRNA of TNF receptor-associated factor 1 (TRAF1) (Figure 3; Table S6), which is reported to bind to TNFR2 (Rothe et al., 1994).

Our RNA-seq data also revealed an increased abundance of BCL3 and ICAM1 transcripts only in *R. conorii*-infected cells (Figure 3; Table S6). BCL3 (B-Cell lymphoma 3-encoded protein) has been documented as a regulator of classical and non-canonical NF-κB-dependent gene transcription and it can limit pro-inflammatory transcriptional programs (Herrington and Nibbs, 2016). On the other hand, ICAM1 (intercellular adhesion molecule-1) is a transmembrane glycoprotein reported to be upregulated in response to different inflammatory mediators and playing a role in immune surveillance (Usami et al., 2013).

Cytokine signaling through Janus kinase (Jak)-signal transducer and activator of transcription (STAT) pathway (Jak-STAT pathway) also has an important role in the control of immune responses (Shuai and Liu, 2003; Villarino et al., 2017). mRNA for six genes categorized to this signaling process were found in increased abundance only in cells infected with *R. conorii* (Table S8).

The overall prediction of inflammatory response in *R. conorii*- and *R. montanensis*-challenged THP-1 macrophages was then evaluated using the downstream “Diseases and Functions” tool provided by IPA (Table 3). The contribution of DE genes associated with this response in *R. conorii*-infected cells resulted in a balance between pro- and anti-inflammatory signals with a predicted null *Z*-score of activation/inhibition (*Z*-score of −0.092; *p*-value of 5.29 × 10^−20^). Transcripts for antimicrobial enzymes, such as cathepsin G (CTSG), elastase (ELANE), and proteinase 3 (PRTN3), that are part of the earliest line of host inflammatory responses against pathogens (Korkmaz et al., 2010; Hahn et al., 2011), were among those found in decreased abundance, predicted to contribute to inhibit inflammatory responses in *R. conorii-*infected cells. Conversely, in *R. montanensis*-infected cells there was a predicted activation of the inflammatory response (*Z*-score of 2.664; *p*-value of 1.01 × 10^−10^). Together, these results anticipate significant differences in inflammatory signaling promoted by these bacterial species, suggesting that the pathogenic *R. conorii* appears to switch immune signals into a hyporesponsive state.

**Table 3:** Predicted contribution of DE genes in *R. conorii*- and *R. montanensis*-THP-1 macrophages for inflammatory response based on “Diseases and Functions” category by IPA.

**Gene I.D**.	**Gene description**	**Prediction**	**Log_**2**_ fold change (*R.con*/Uninf.)**
**THP-1 MACROPHAGES INFECTED WITH *R. conorii***			
ACKR3	Atypical chemokine receptor 3(ACKR3)	Inhibition	0.81
ADGRE5	Adhesion G protein-coupled receptor E5(ADGRE5)	Affected	−0.66
AGT	Angiotensinogen(AGT)	Inhibition	−0.57
ATF3	Activating transcription factor 3(ATF3)	Inhibition	3.08
CCL20	C-C motif chemokine ligand 20(CCL20)	Inconsistent	4.57
CCL3	C-C motif chemokine ligand 3(CCL3)	Inconsistent	5.55
CCL3L3	C-C motif chemokine ligand 3 like 3(CCL3L3)	Affected	7.05
CCR1	C-C motif chemokine receptor 1(CCR1)	Inhibition	−0.56
CD14	CD14 molecule(CD14)	Inhibition	−0.86
CD69	CD69 molecule(CD69)	Inhibition	3.71
CDO1	Cysteine dioxygenase type 1(CDO1)	Affected	−1.82
CTSG	Cathepsin G(CTSG)	Inhibition	−0.69
CXCL1	C-X-C motif chemokine ligand 1(CXCL1)	Inconsistent	5.59
CXCL3	C-X-C motif chemokine ligand 3(CXCL3)	Inconsistent	5.55
CXCL8	C-X-C motif chemokine ligand 8(CXCL8)	Inconsistent	4.24
EDN1	Endothelin 1(EDN1)	Inconsistent	2.15
ELANE	Elastase, neutrophil expressed(ELANE)	Inhibition	−0.71
ENG	Endoglin(ENG)	Inconsistent	−0.75
FOS	Fos proto-oncogene, AP-1 transcription factor subunit(FOS)	Affected	1.21
ICAM1	Intercellular adhesion molecule 1(ICAM1)	Inconsistent	1.63
IL1A	Interleukin 1 alpha(IL1A)	Inconsistent	5.59
IL1B	Interleukin 1 beta(IL1B)	Inconsistent	2.98
LGALS3BP	Galectin 3 binding protein(LGALS3BP)	Inconsistent	−0.61
LPAR2	Lysophosphatidic acid receptor 2(LPAR2)	Inhibition	−0.89
MCL1	BCL2 family apoptosis regulator(MCL1)	Inhibition	0.78
MIR223	microRNA 223(MIR223)	Inconsistent	−0.89
NFE2L2	Nuclear factor, erythroid 2 like 2(NFE2L2)	Inhibition	1.28
NFIL3	Nuclear factor, interleukin 3 regulated(NFIL3)	Affected	0.47
NFKBIA	NFKB inhibitor alpha(NFKBIA)	Inconsistent	5.29
NLRP3	NLR family pyrin domain containing 3(NLRP3)	Inhibition	1.14
NOTCH1	Notch 1(NOTCH1)	Inhibition	−0.70
OSM	Oncostatin M(OSM)	Affected	3.99
PDGFB	Platelet derived growth factor subunit B(PDGFB)	Affected	3.10
PLAU	Plasminogen activator, urokinase(PLAU)	Inconsistent	1.45
PRTN3	Proteinase 3(PRTN3)	Inhibition	−0.73
PTGER4	prostaglandin E receptor 4(PTGER4)	Inhibition	1.55
PTGS2	Prostaglandin-endoperoxide synthase 2(PTGS2)	Inconsistent	4.86
PTX3	Pentraxin 3(PTX3)	Affected	1.75
RGS1	Regulator of G-protein signaling 1(RGS1)	Inhibition	1.05
RIPK2	Receptor interacting serine/threonine kinase 2(RIPK2)	Affected	1.32
SCARB1	Scavenger receptor class B member 1(SCARB1)	Inhibition	−0.59
SCG2	Secretogranin II(SCG2)	Inhibition	−0.99
SDC4	Syndecan 4(SDC4)	Affected	0.78
SMAD7	SMAD family member 7(SMAD7)	Inhibition	0.85
SOCS3	Suppressor of cytokine signaling 3(SOCS3)	Inhibition	4.89
TICAM1	Toll like receptor adaptor molecule 1(TICAM1)	Affected	0.53
TNF	Tumor necrosis factor(TNF)	Inconsistent	7.93
TUBB2A	Tubulin beta 2A class IIa(TUBB2A)	Affected	1.44
ZFP36	ZFP36 ring finger protein(ZFP36)	Affected	2.76
**THP-1 MACROPHAGES INFECTED WITH *R. montanensis***			
ATF3	Activating transcription factor 3(ATF3)	Inconsistent	1.34
CCL20	C-C motif chemokine ligand 20(CCL20)	Activation	2.97
CCL3	C-C motif chemokine ligand 3(CCL3)	Activation	3.78
CCL3L3	C-C motif chemokine ligand 3 like 3(CCL3L3)	Affected	5.08
CD69	CD69 molecule(CD69)	Inconsistent	2.20
CXCL1	C-X-C motif chemokine ligand 1(CXCL1)	Activation	3.84
CXCL3	C-X-C motif chemokine ligand 3(CXCL3)	Activation	3.35
CXCL8	C-X-C motif chemokine ligand 8(CXCL8)	Activation	2.95
EDN1	Endothelin 1(EDN1)	Activation	1.62
IL1B	Interleukin 1 beta(IL1B)	Activation	1.41
NFKBIA	NFKB inhibitor alpha(NFKBIA)	Activation	3.16
PTGS2	Prostaglandin-endoperoxide synthase 2(PTGS2)	Activation	2.74
TNF	Tumor necrosis factor(TNF)	Activation	5.34
TNFSF11	Tumor necrosis factor superfamily member 11(TNFSF11)	Affected	2.12
ZFP36	ZFP36 ring finger protein(ZFP36)	Affected	0.76

*The software predicted activation/inhibition Z-scores of −0.092 (p-value of 5.29 × 10^−20^) and 2.664 (p-value of 1.01 × 10^−10^) for R. conorii- and R. montanensis-infected THP-1 macrophages, respectively. In IPA, Z-scores are used to predict activation or inhibition, where a Z-score ≥ 2.0 represents a statistical significant result of activation while a Z-score ≤ 2.0 yields a prediction of inhibition. Individual genes are color coded according to their contribution for the overall prediction: orange-leads to activation; blue-leads to inhibition; yellow-findings are inconsistent with the state of downstream function; black-effect is not predicted*.

### *Rickettsia conorii* Blocks Secretion of TNFα Stimulated by LPS

**Figure 4:**
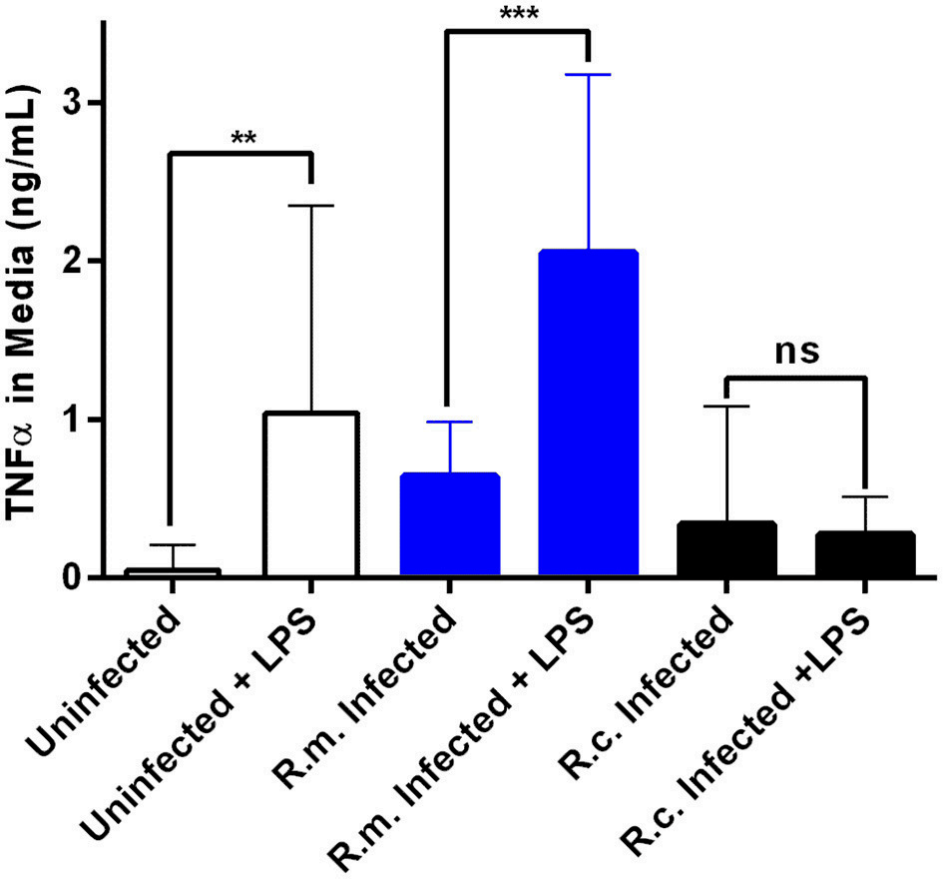
*Rickettsia conorii* switches macrophage immune responses into a hyporesponsive state. Quantification of TNFα concentration in the culture media of uninfected (white), *R. conorii*- (black) and *R. montanensis*-infected (blue) THP-1 macrophages upon stimulation with *E. coli* O26:B6 LPS. Results are shown as mean ± SD and differences were considered non-significant (ns) at *P* > 0.05 or significant at **P ≤ 0.01, ***P ≤ 0.001.

To further characterize the anticipated differences in inflammatory response between *R. conorii* and *R. montanensis*-infected cells, we next evaluated how uninfected and infected THP-1 cells respond to a well-known pro-inflammatory stimulus (*E. coli* O26:B6 LPS) 24 h after infection (Figure 4). As expected, uninfected THP-1 cells responded by increasing secretion of TNFα and a similar result was obtained in *R. montanensis*-infected cells. However, in sharp contrast *R. conorii*-infected cells were unresponsive to LPS stimulation, displaying levels of secreted TNFα comparable to non-stimulated cells (Figure 4). Overall, these results suggest that the pathogenic *R. conorii* can modulate TNFα signaling in macrophages which likely impacts regulation of inflammatory cell activation.

### *Rickettsia conorii* Actively Modulates Pro-survival Pathways to Sustain Macrophage Viability During Infection

**Figure 5:**
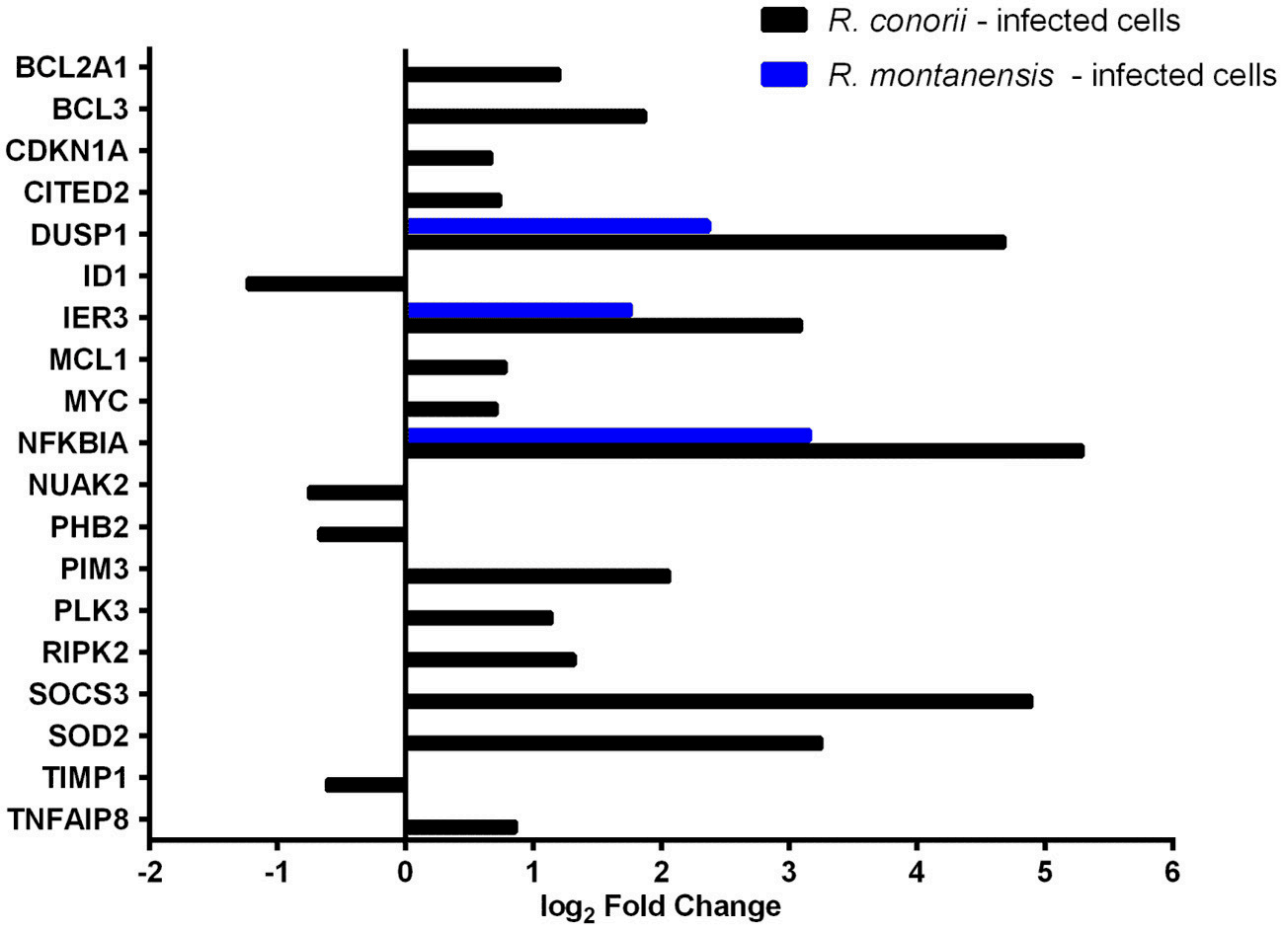
*Rickettsia* species differentially modulate the expression of several host apoptotic genes early in infection of THP-1 macrophages. Log_2_ fold change values of DE genes categorized with GO term “negative regulation of apoptotic process” (GO:0043066) in *R. conorii*- (black) and *R. montanensis*-infected (blue) cells. Absence of bar means that the fold change of that gene for the respective experimental condition was not considered statistically significant. See also Table S9.

Apoptosis is part of the arsenal of defense mechanisms used to prevent infection. However, pathogens themselves have evolved numerous ways to modulate cell death pathways, and intracellular microorganisms can subvert nearly all steps of the apoptotic cascade (Gao and Kwaik, 2000; Friedrich et al., 2017). THP-1 cells infected with *R. conorii* showed a striking difference in the number of DE genes grouped to the negative regulation of the apoptotic process (GO:0043066), with 16 out of the 19 DE genes showing altered abundance in *R. conorii*-infected cells only (Figure 5; Table S9). Among these genes were MCL1 and BCL2A1, two Bcl-2 protein family members known as important regulators of the integrity of mitochondria (Willis and Adams, 2005), PIM3 that can prevent apoptosis and promote cell survival (Mukaida et al., 2011), and the mitochondrial protein superoxide dismutase 2 (SOD2) involved in protection against oxidative stress (Drane et al., 2001). Furthermore, IPA “Diseases and functions” downstream analysis identified the contribution of 73 DE genes for cell survival in cells infected with *R. conorii* (predicted activation *Z*-score of 3.661; *p*-value of 1.41 × 10^−15^), against the non-significant *Z*-score of 1.960; *p*-value of 6.11 × 10^−9^ retrieved for *R. montanensis*-challenged cells (Table 4). Globally, our results showed an *R. conorii*-specific increase in abundance of several transcripts encoding products with important functions in the control of host cell survival and modulation of responses against inflammatory cytokines, further reinforcing the trend already observed with other pro-survival genes (GADD45B, TRAF1, BCL2A1, CXCL8, and PLAU). These results are consistent with our previously reported phenotypic differences, supporting the ability of *R. conorii* to establish a niche in THP-1 macrophages while the integrity of *R. montanensis*-infected cells was shown to be compromised (Curto et al., 2016).

**Table 4:** Predicted contribution of DE genes in *R. conorii*- and *R. montanensis*-THP-1 macrophages for cell survival based on “Diseases and Functions” category by IPA.

**Gene I.D**.	**Gene description**	**Prediction**	**Log_**2**_ fold change (*R.con*/Uninf.)**
**THP-1 MACROPHAGES INFECTED WITH *R. conorii***			
ABCB6	ATP binding cassette subfamily B member 6 (Langereis blood group)(ABCB6)	Inconsistent	−0.55
ACKR3	Atypical chemokine receptor 3(ACKR3)	Activation	0.81
ATF3	Activating transcription factor 3(ATF3)	Inconsistent	3.08
BCL2A1	BCL2 related protein A1(BCL2A1)	Activation	1.2
BCL3	B-cell CLL/lymphoma 3(BCL3)	Activation	1.87
BCL6	B-cell CLL/lymphoma 6(BCL6)	Affected	1.1
BTG2	BTG anti-proliferation factor 2(BTG2)	Activation	2.97
CCL3	C-C motif chemokine ligand 3(CCL3)	Activation	5.55
CCR1	C-C motif chemokine receptor 1(CCR1)	Affected	−0.56
CDKN1A	Cyclin dependent kinase inhibitor 1A(CDKN1A)	Activation	0.67
CEBPD	CCAAT/enhancer binding protein delta(CEBPD)	Activation	0.62
CTGF	Connective tissue growth factor(CTGF)	Activation	2.59
CXCL1	C-X-C motif chemokine ligand 1(CXCL1)	Activation	5.59
CXCL3	C-X-C motif chemokine ligand 3(CXCL3)	Activation	5.55
CXCL8	C-X-C motif chemokine ligand 8(CXCL8)	Activation	4.24
DUSP1	Dual specificity phosphatase 1(DUSP1)	Activation	4.68
DUSP10	Dual specificity phosphatase 10(DUSP10)	Activation	0.77
DUSP5	Dual specificity phosphatase 5(DUSP5)	Activation	0.96
ECM1	Extracellular matrix protein 1(ECM1)	Inconsistent	−0.74
EDN1	Endothelin 1(EDN1)	Inconsistent	2.15
ELANE	Elastase, neutrophil expressed(ELANE)	Activation	−0.71
ENG	Endoglin(ENG)	Inconsistent	−0.75
EPHB2	EPH receptor B2(EPHB2)	Affected	−0.77
ETS2	ETS proto-oncogene 2, transcription factor(ETS2)	Activation	1.07
FOS	Fos proto-oncogene, AP-1 transcription factor subunit(FOS)	Activation	1.21
FOSL1	FOS like 1, AP-1 transcription factor subunit(FOSL1)	Activation	1.01
FTH1	Ferritin heavy chain 1(FTH1)	Activation	1.04
ICAM1	Intercellular adhesion molecule 1(ICAM1)	Activation	1.63
IER3	Immediate early response 3(IER3)	Inconsistent	3.09
IL1A	Interleukin 1 alpha(IL1A)	Activation	5.59
IL1B	Interleukin 1 beta(IL1B)	Inconsistent	2.98
JUN	JUN proto-oncogene, AP-1 transcription factor subunit(JUN)	Activation	3.14
KDM6B	Lysine demethylase 6B(KDM6B)	Inconsistent	2.17
KLF2	Kruppel like factor 2(KLF2)	Activation	2.5
KLF6	Kruppel like factor 6(KLF6)	Activation	1.68
LGALS3BP	Galectin 3 binding protein(LGALS3BP)	Inconsistent	−0.61
LPAR2	Lysophosphatidic acid receptor 2(LPAR2)	Activation	−0.89
MCL1	BCL2 family apoptosis regulator(MCL1)	Activation	0.78
MIR137	microRNA 137(MIR137)	Inconsistent	2.13
MIR223	microRNA 223(MIR223)	Activation	−0.89
MRM1	Mitochondrial rRNA methyltransferase 1(MRM1)	Inconsistent	−0.71
MYC	v-myc avian myelocytomatosis viral oncogene homolog(MYC)	Activation	0.71
NAMPT	Nicotinamide phosphoribosyltransferase(NAMPT)	Activation	1.25
NFE2L2	Nuclear factor, erythroid 2 like 2(NFE2L2)	Activation	1.28
NFIL3	Nuclear factor, interleukin 3 regulated(NFIL3)	Activation	0.47
NFKB2	Nuclear factor kappa B subunit 2(NFKB2)	Activation	1.1
NFKBIA	NFKB inhibitor alpha(NFKBIA)	Inconsistent	5.29
NOTCH1	Notch 1(NOTCH1)	Inconsistent	−0.7
NUP210	Nucleoporin 210(NUP210)	Inconsistent	−0.59
OSM	Oncostatin M(OSM)	Activation	3.99
PDGFB	Platelet derived growth factor subunit B(PDGFB)	Affected	3.1
PHB2	prohibitin 2(PHB2)	Affected	−0.67
PIM3	Pim-3 proto-oncogene, serine/threonine kinase(PIM3)	Activation	2.06
PLAU	Plasminogen activator, urokinase(PLAU)	Activation	1.45
PMAIP1	Phorbol-12-myristate-13-acetate-induced protein 1(PMAIP1)	Inconsistent	1.02
PTGS2	Prostaglandin-endoperoxide synthase 2(PTGS2)	Activation	4.86
REL	REL proto-oncogene, NF-kB subunit(REL)	Activation	0.66
RET	Ret proto-oncogene(RET)	Inconsistent	−0.6
RIPK2	Receptor interacting serine/threonine kinase 2(RIPK2)	Activation	1.32
SAT1	Spermidine/spermine N1-acetyltransferase 1(SAT1)	Inconsistent	1.5
SGK1	Serum/glucocorticoid regulated kinase 1(SGK1)	Activation	0.99
SNAI1	Snail family transcriptional repressor 1(SNAI1)	Activation	2.75
SOCS3	SUPPRESSOR of cytokine signaling 3(SOCS3)	Activation	4.89
SOD2	superoxide dismutase 2, mitochondrial(SOD2)	Activation	3.25
TICAM1	Toll like receptor adaptor molecule 1(TICAM1)	Activation	0.53
TIMP1	TIMP metallopeptidase inhibitor 1(TIMP1)	Inconsistent	−0.61
TIMP3	TIMP metallopeptidase inhibitor 3(TIMP3)	Activation	−0.63
TNF	Tumor necrosis factor(TNF)	Inconsistent	7.93
TNFAIP3	TNF alpha induced protein 3(TNFAIP3)	Activation	3.68
TNFAIP8	TNF alpha induced protein 8(TNFAIP8)	Activation	0.86
TNFSF9	Tumor necrosis factor superfamily member 9(TNFSF9)	Activation	1.25
TUBGCP6	Tubulin gamma complex associated protein 6(TUBGCP6)	Inconsistent	−0.53
ZFP36	ZFP36 ring finger protein(ZFP36)	Inconsistent	2.76
**THP-1 MACROPHAGES INFECTED WITH *R. montanensis***			
ATF3	Activating transcription factor 3(ATF3)	Inconsistent	1.34
BTG2	BTG anti-proliferation factor 2(BTG2)	Activation	1.07
CCL3	C-C motif chemokine ligand 3(CCL3)	Activation	3.78
CTGF	Connective tissue growth factor(CTGF)	Activation	1.21
CXCL1	C-X-C motif chemokine ligand 1(CXCL1)	Activation	3.84
CXCL3	C-X-C motif chemokine ligand 3(CXCL3)	Activation	3.35
CXCL8	C-X-C motif chemokine ligand 8(CXCL8)	Activation	2.95
DUSP1	Dual specificity phosphatase 1(DUSP1)	Activation	2.37
EDN1	Endothelin 1(EDN1)	Inconsistent	1.62
IER3	Immediate early response 3(IER3)	Inconsistent	1.76
IL1B	Interleukin 1 beta(IL1B)	Inconsistent	1.41
JUN	Jun proto-oncogene, AP-1 transcription factor subunit(JUN)	Activation	1.35
KLF5	Kruppel like factor 5(KLF5)	Activation	3.65
NFKBIA	NFKB inhibitor alpha(NFKBIA)	Inconsistent	3.16
PTGS2	Prostaglandin-endoperoxide synthase 2(PTGS2)	Activation	2.74
SLC40A1	Solute carrier family 40 member 1(SLC40A1)	Inconsistent	−1.92
TNF	Tumor necrosis factor(TNF)	Inconsistent	5.34
TNFAIP3	TNF alpha induced protein 3(TNFAIP3)	Activation	2.08
TNFSF11	Tumor necrosis factor superfamily member 11(TNFSF11)	Activation	2.12
ZFP36	ZFP36 ring finger protein(ZFP36)	Inconsistent	0.76

*The software predicted activation/inhibition Z-scores of 3.661 (p-value of 1.41 × 10^−15^) and 1.960 (p-value of 6.11 × 10^−9^) for R. conorii- and R. montanensis-infected THP-1 macrophages, respectively. In IPA, Z-scores are used to predict activation or inhibition, where a Z-score ≥ 2.0 represents a statistical significant result of activation while a Z-score ≤ 2.0 yields a prediction of inhibition. Individual genes are color coded according to their contribution for the overall prediction: orange-leads to activation; yellow-findings are inconsistent with the state of downstream function; black-effect is not predicted*.

**Figure 6:**
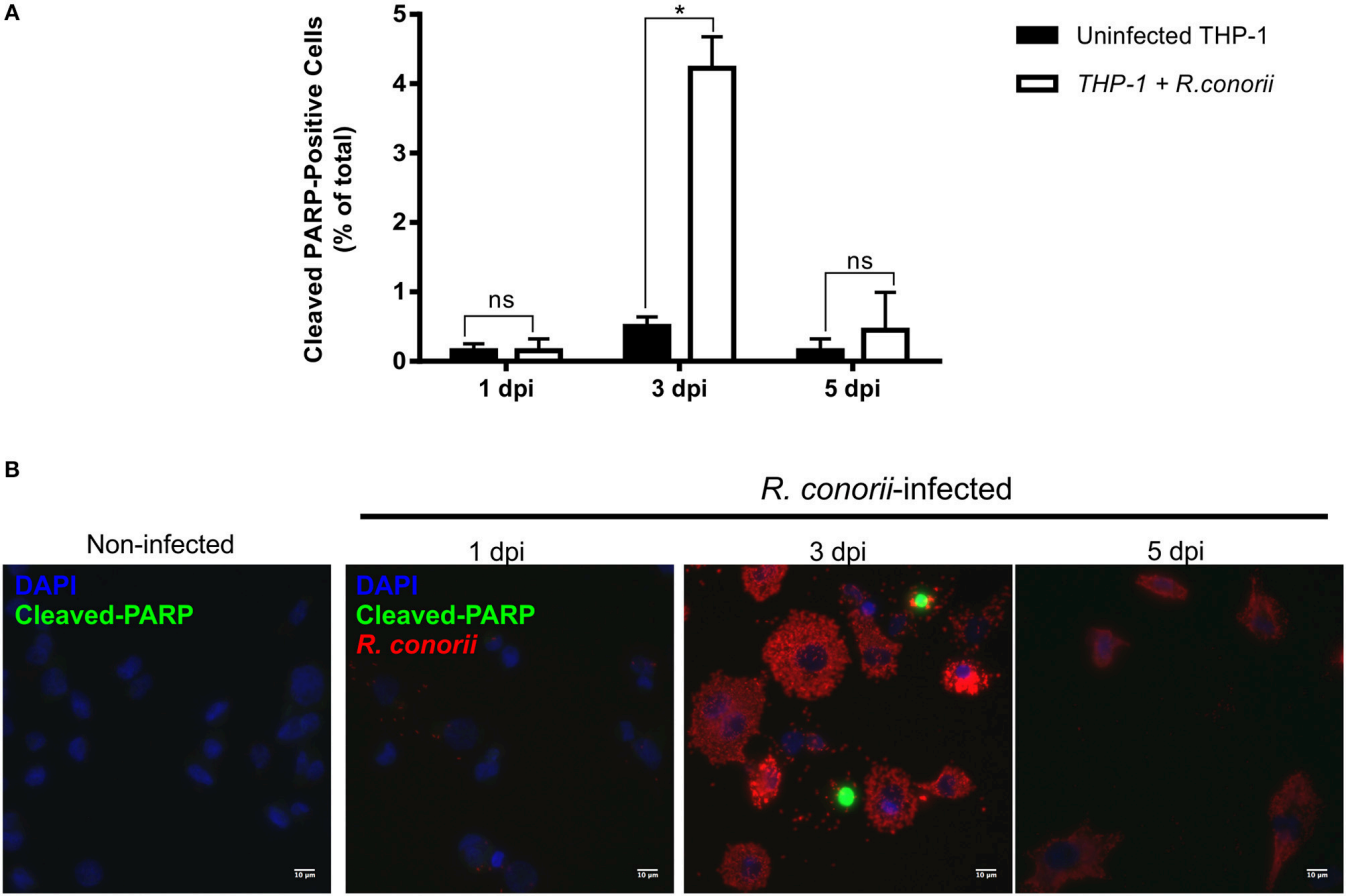
*Rickettsia conorii* modulates host cell apoptosis during infection. **(A)** Percentage of cleaved PARP-positive cells, a marker of intrinsic apoptosis, over the course of infection of THP-1 macrophages with *R. conorii*. Results are shown as the mean ± SD and differences were considered non-significant (ns) at *P* > 0.05 or significant at ^*^P < 0.05. **(B)** Immunofluorescence microscopy of uninfected cells and THP-1 macrophages infected with *R. conorii* at 1, 3, and 5 days post-infection. Cells were stained with DAPI (blue) to identify host nuclei, mouse anti-*Rickettsia* antibody (5C7.31) followed by anti-mouse Alexa Fluor 594 (red) to identify *R. conorii* and rabbit anti-cleaved poly(ADP-ribose) polymerase (PARP) followed by anti-rabbit Alexa Fluor 488 (green) to identify cleaved PARP-positive cells. Scale bar = 10 μm.

**Figure 7:**
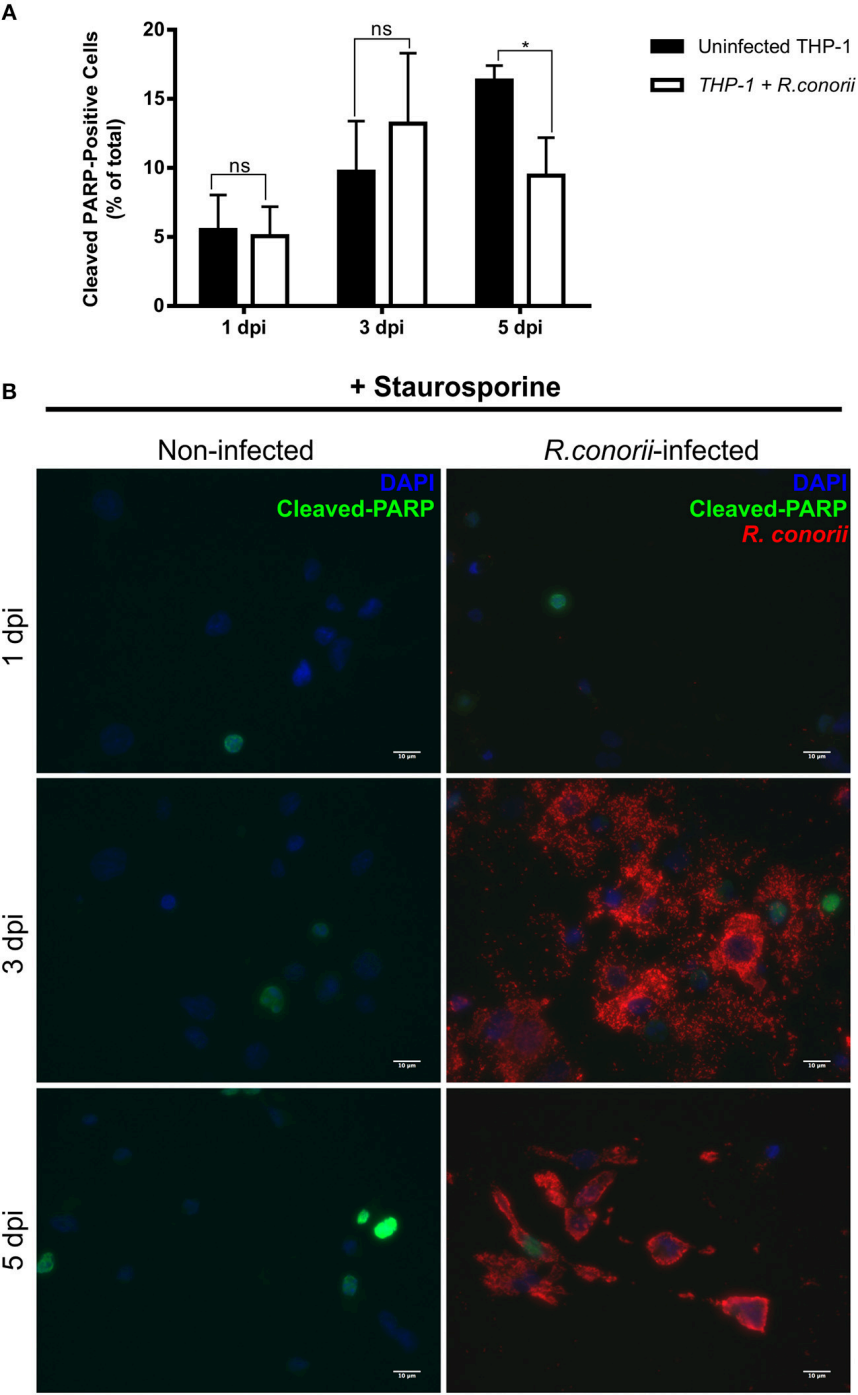
*Rickettsia conorii* inhibits staurosporine-induced death of THP-1 macrophages at 5 days post-infection. **(A)** Uninfected and *R. conorii*-infected THP-1 macrophages at 1, 3, and 5 days post-infection were treated with staurosporine (750 nM) for 4 h. The percentage of cleaved PARP-positive cells in each experimental condition is shown as the mean ± SD and differences were considered ns (non-significant) at *P* > 0.05 or significant at ^*^P < 0.05. **(B)** Immunofluorescence microscopy of uninfected cells and THP-1 macrophages infected with *R. conorii* at 1, 3, and 5 days post-infection upon challenge with staurosporine (750 nM) for 4 h. Cells were stained with DAPI (blue) to identify host nuclei, mouse anti-*Rickettsia* antibody (5C7.31) followed by anti-mouse Alexa Fluor 594 (red) to identify *R. conorii* and rabbit anti-cleaved poly(ADP-ribose) polymerase (PARP) followed by anti-rabbit Alexa Fluor 488 (green) to identify cleaved PARP-positive cells. Scale bar = 10 μm.

Previous findings on infection of endothelial cells with *R. rickettsii* revealed no effect on poly(ADP-ribose) polymerase (PARP-1) cleavage (a classical marker of the terminal stages of apoptosis) for up to 18 h after infection (Joshi et al., 2003). To further evaluate if the pro-survival manipulation of the host cell by *R. conorii* is maintained over the course of the infection, we quantified cleaved PARP-1, in both uninfected and *R. conorii*-infected THP-1 macrophages by immunofluorescence microscopy analysis (IFA), at 24, 72, and 120 h post-infection (Figure 6). We observed no significant increase in the number of cleaved PARP-positive cells at 24 and 120 h post-infection, although a significant increase in this number was found at the intermediate time-point (72 h), suggesting a controlled (but dynamic) modulation of host cell apoptosis by *R. conorii*. We then evaluated if infection with *R. conorii* was able to protect these cells from treatment with a potent inducer of intrinsic apoptosis, staurosporine. Our results showed a significant reduction in cleaved PARP-positive THP-1 cells at 120 h post-infection when compared with uninfected control cells, confirming protection from staurosporine-induced apoptosis triggered by *R. conorii* infection (Figure 7). The altered expression of several genes with important functions as negative regulators of apoptosis as early as 1 h post-infection, combined with these findings for longer time-points post infection suggest that *R. conorii* actively modulates apoptotic signaling to sustain the viability of the host cell.

### SFG *Rickettsia* Species Promote Robust Changes in Expression of Several Gene Expression Regulators Early in Infection of THP-1 Macrophages

**Figure 8:**
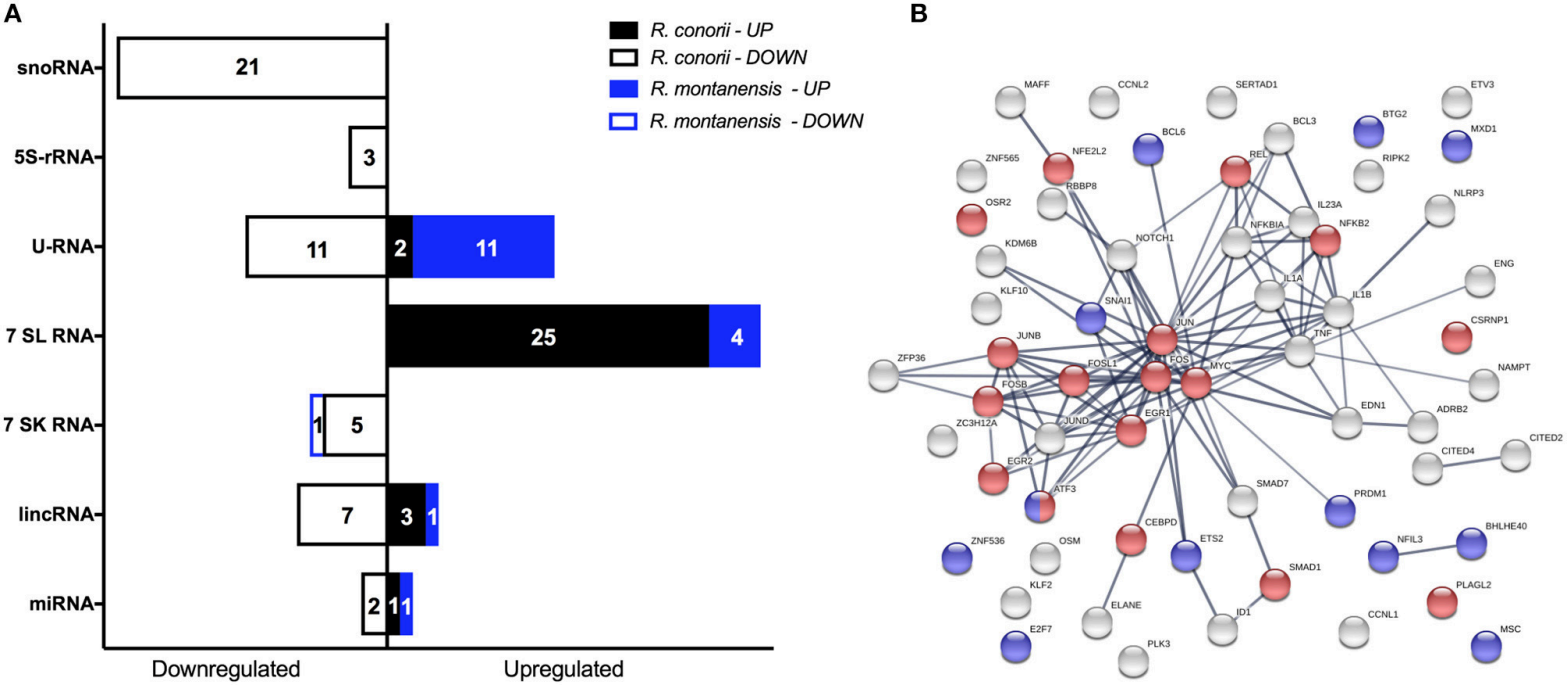
*Rickettsia* species differentially modulate the expression of several gene expression regulators early in infection of THP-1 macrophages. **(A)** Distribution of DE non-coding RNAs according to their category in *R. conorii*- (black) and *R. montanensis*- (blue) infected cells. scaRNAs (small Cajal body-specific RNAs), snoRNAs (small nucleolar RNAs), 5S-rRNAs (5S ribosomal RNAs), U-RNA (small nuclear RNAs), 7SL RNAs (signal recognition particle RNAs), 7SK RNAs (7SK small nuclear RNAs), lincRNAs (long intergenic noncoding RNAs), miRNAs (microRNAs). Number of genes for each orientation [increased abundance (UP) or decreased abundance (DOWN)] is represented in each bar. See Table S10 for details about individual transcripts. **(B)** STRING analysis of DE genes in *R. conorii*-infected cells categorized in the GO term “positive or negative regulators of transcription from RNA polymerase II promoter.” Nodes corresponding to DE genes categorized with transcriptional activator activity (GO:0001228) are in red and with transcriptional repressor activity (GO:0001227) are in blue. See also Table S11.

Recognition of infectious agents by host cells results in alterations of transcriptional programs in order tackle the infection (Asrat et al., 2015). However, it is now becoming clear that pathogens can reprogram host gene expression profiles by directly targeting or altering these programs at the level of transcriptional regulation (Bierne and Cossart, 2012; Asrat et al., 2015). In addition to protein coding transcripts, it was also possible to identify several ncRNAs differentially expressed within our datasets. Infection with each SFG *Rickettsia* resulted not only in a robust response regarding these regulatory elements, with multiple ncRNAs of different types being DE at 1 h post-infection, but also in a very different pattern of alterations between rickettsial species (Figure 8A; Table S10). Overall, 80 ncRNAs were found altered in *R. conorii*-infected cells, whereas only 18 were observed in *R. montanensis*-infected cells (Figure 8A).

To evaluate potential differences in pathways involved in transcriptional regulation between our data sets, we utilized the “Diseases and Functions” downstream analysis on IPA (Table 5). Under both experimental conditions, transcription was predicted to be activated upon infection (*R. conorii*-infected: *Z*-score of 2.428 and *p*-value of 5.27 × 10^−14^; *R. montanensis*-infected: *Z*-score of 2.647 and *p*-value of 7.48 × 10^−6^). However, the number of genes predicted to impact this process differed substantially between infected conditions (Table 5). In cells infected with *R. conorii*, 81 DE genes were predicted to affect transcription whereas only 18 DE genes were associated with this process in *R. montanensis*-infected dataset (Table 5). Moreover, a large number of these DE genes in the *R. conorii* dataset (61 genes) were categorized as either positive or negative regulators of transcription from RNA polymerase II (RNAP II) promoter (Table S11). We further analyzed the potential relationships among these 61 DE genes using the STRING database (Figure 8B). Of these regulators, 27 genes were categorized as transcription factors involved in positive regulation (nodes in red) and in negative regulation of transcription (nodes in blue) (RNA polymerase II transcription regulatory region sequence-specific DNA binding: GO:0001228—Transcriptional activator activity; GO:0001227—Transcriptional repressor activity). Moreover, members of the AP-1 transcription factor complex appear as central nodes in this interaction network (Figure 8B). Together with the more substantial reprograming globally observed in *R. conorii*-infected cells (409 DE genes, Figure 2A), this robust alteration of different transcription factors by *R. conorii* further suggests that modification of the transcriptional machinery early in infection might be critical to prolonging host survival and, as a result, bacterial survival and proliferation in THP-1 macrophages.

**Table 5:** Predicted contribution of DE genes in *R. conorii*- and *R. montanensis*-THP-1 macrophages for transcription based on “Diseases and Functions” category by IPA.

**Gene I.D**.	**Gene description**	**Prediction**	**Log_**2**_ fold change (*R.con*/Uninf.)**
**THP-1 MACROPHAGES INFECTED WITH *R. conorii***			
AGT	Angiotensinogen(AGT)	Inconsistent	−0.57
ATF3	Activating transcription factor 3(ATF3)	Affected	3.08
BCL3	B-cell CLL/lymphoma 3(BCL3)	Inconsistent	1.87
BCL6	B-cell CLL/lymphoma 6(BCL6)	Inconsistent	1.1
BHLHE40	Basic helix-loop-helix family member e40(BHLHE40)	Inconsistent	2.49
BTG2	BTG anti-proliferation factor 2(BTG2)	Activation	2.97
CCL3	C-C motif chemokine ligand 3(CCL3)	Affected	5.55
CCR1	C-C motif chemokine receptor 1(CCR1)	Activation	−0.56
CDKN1A	Cyclin dependent kinase inhibitor 1A(CDKN1A)	Inconsistent	0.67
CEBPD	CCAAT/enhancer binding protein delta(CEBPD)	Affected	0.62
CITED2	Cbp/p300 interacting transactivator with Glu/Asp rich Carboxy-terminal domain 2(CITED2)	Affected	0.74
CREB5	cAMP responsive element binding protein 5(CREB5)	Activation	2.65
CSRNP1	Cysteine and serine rich nuclear protein 1(CSRNP1)	Activation	1.75
CXCL3	C-X-C motif chemokine ligand 3(CXCL3)	Activation	5.55
E2F7	E2F transcription factor 7(E2F7)	Affected	0.78
ECM1	Extracellular matrix protein 1(ECM1)	Inconsistent	−0.74
EDN1	Endothelin 1(EDN1)	Affected	2.15
EGR1	Early growth response 1(EGR1)	Activation	3.32
EGR2	Early growth response 2(EGR2)	Activation	0.91
EGR4	Early growth response 4(EGR4)	Activation	3.94
ELANE	Elastase, neutrophil expressed(ELANE)	Activation	−0.71
ENG	endoglin(ENG)	Affected	−0.75
ETS2	ETS proto-oncogene 2, transcription factor(ETS2)	Activation	1.07
FOS	Fos proto-oncogene, AP-1 transcription factor subunit(FOS)	Activation	1.21
FOSB	FosB proto-oncogene, AP-1 transcription factor subunit(FOSB)	Activation	4.02
FOSL1	FOS like 1, AP-1 transcription factor subunit(FOSL1)	Activation	1.01
H1F0	H1 histone family member 0(H1F0)	Inconsistent	0.53
ID1	Inhibitor of DNA binding 1, HLH protein(ID1)	Activation	−1.23
IER2	Immediate early response 2(IER2)	Activation	1.54
IER5	Immediate early response 5(IER5)	Activation	1.37
IL1A	Interleukin 1 alpha(IL1A)	Activation	5.59
IL1B	Interleukin 1 beta(IL1B)	Activation	2.98
JUN	Jun proto-oncogene, AP-1 transcription factor subunit(JUN)	Activation	3.14
KDM6B	Lysine demethylase 6B(KDM6B)	Activation	2.17
KLF2	Kruppel like factor 2(KLF2)	Activation	2.5
KLF6	Kruppel like factor 6(KLF6)	Activation	1.68
KLF10	Kruppel like factor 10(KLF10)	Activation	2.4
LCP2	Lymphocyte cytosolic protein 2(LCP2)	Affected	0.65
LPAR2	Lysophosphatidic acid receptor 2(LPAR2)	Activation	−0.89
MAFF	MAF bZIP transcription factor F(MAFF)	Activation	1.74
MID1IP1	MID1 interacting protein 1(MID1IP1)	Activation	0.51
MIR223	microRNA 223(MIR223)	Inconsistent	−0.89
MSC	Musculin(MSC)	Affected	0.89
MXD1	MAX dimerization protein 1(MXD1)	Inconsistent	0.81
MYC	v-myc avian myelocytomatosis viral oncogene homolog(MYC)	Activation	0.71
NAMPT	Nicotinamide phosphoribosyltransferase(NAMPT)	Activation	1.25
NFE2L2	Nuclear factor, erythroid 2 like 2(NFE2L2)	Activation	1.28
NFIL3	Nuclear factor, interleukin 3 regulated(NFIL3)	Inconsistent	0.47
NFKB2	Nuclear factor kappa B subunit 2(NFKB2)	Affected	1.10
NFKBIA	NFKB inhibitor alpha(NFKBIA)	Activation	5.29
NFKBIZ	NFKB inhibitor zeta(NFKBIZ)	Affected	4.53
NOCT	Nocturnin(NOCT)	Affected	1.66
NOTCH1	Notch 1(NOTCH1)	Activation	−0.70
OSM	Oncostatin M(OSM)	Activation	3.99
PCDH11Y	Protocadherin 11 Y-linked(PCDH11Y)	Inconsistent	−4.00
PDGFB	Platelet derived growth factor subunit B(PDGFB)	Affected	3.10
PHB2	Prohibitin 2(PHB2)	Activation	−0.67
PLK3	Polo like kinase 3(PLK3)	Inconsistent	1.14
PRDM1	PR/SET domain 1(PRDM1)	Inconsistent	2.05
PTGER4	Prostaglandin E receptor 4(PTGER4)	Activation	1.55
PTGES3	Prostaglandin E synthase 3(PTGES3)	Affected	0.59
RBBP8	RB binding protein 8, endonuclease(RBBP8)	Activation	−2.55
REL	REL proto-oncogene, NF-kB subunit(REL)	Activation	0.66
RET	Ret proto-oncogene(RET)	Inconsistent	−0.60
RHOB	Ras homolog family member B(RHOB)	Activation	1.31
RIPK2	Receptor interacting serine/threonine kinase 2(RIPK2)	Activation	1.32
SGK1	Serum/glucocorticoid regulated kinase 1(SGK1)	Inconsistent	0.99
SMAD1	SMAD family member 1(SMAD1)	Inconsistent	−1.75
SMAD7	SMAD family member 7(SMAD7)	Inconsistent	0.85
SNAI1	Snail family transcriptional repressor 1(SNAI1)	Inconsistent	2.75
SOCS3	Suppressor of cytokine signaling 3(SOCS3)	Inconsistent	4.89
SOD2	Superoxide dismutase 2, mitochondrial(SOD2)	Affected	3.25
TIMP1	TIMP metallopeptidase inhibitor 1(TIMP1)	Activation	−0.61
TIMP3	TIMP metallopeptidase inhibitor 3(TIMP3)	Activation	−0.63
TNF	Tumor necrosis factor(TNF)	Activation	7.93
TRAF1	TNF receptor associated factor 1(TRAF1)	Activation	1.44
ZBTB5	Zinc finger and BTB domain containing 5(ZBTB5)	Inconsistent	0.77
ZC3H12A	Zinc finger CCCH-type containing 12A(ZC3H12A)	Activation	2.39
ZFP36	ZFP36 ring finger protein(ZFP36)	Inconsistent	2.76
ZNF536	Zinc finger protein 536(ZNF536)	Activation	−0.73
ZNF692	Zinc finger protein 692(ZNF692)	Activation	−0.59
**THP-1 MACROPHAGES INFECTED WITH *R. montanensis***			
ATF3	Activating transcription factor 3(ATF3)	Affected	1.34
BTG2	BTG anti-proliferation factor 2(BTG2)	Activation	1.07
CCL3	C-C motif chemokine ligand 3(CCL3)	Affected	3.78
CXCL3	C-X-C motif chemokine ligand 3(CXCL3)	Activation	3.35
EDN1	Endothelin 1(EDN1)	Affected	1.62
EGR1	Early growth response 1(EGR1)	Activation	0.80
IL1B	Interleukin 1 beta(IL1B)	Activation	1.41
JUN	Jun proto-oncogene, AP-1 transcription factor subunit(JUN)	Activation	1.35
KLF5	Kruppel like factor 5(KLF5)	Activation	3.65
KLF10	Kruppel like factor 10(KLF10)	Activation	0.85
NFKBIA	NFKB inhibitor alpha(NFKBIA)	Activation	3.16
NFKBIZ	NFKB inhibitor zeta(NFKBIZ)	Affected	2.78
NR2F2	Nuclear receptor subfamily 2 group F member 2(NR2F2)	Inconsistent	3.69
RBBP8	RB binding protein 8, endonuclease(RBBP8)	Activation	−2.57
TNF	Tumor necrosis factor(TNF)	Activation	5.34
TNFSF11	Tumor necrosis factor superfamily member 11(TNFSF11)	Activation	2.12
ZC3H12A	Zinc finger CCCH-type containing 12A(ZC3H12A)	Activation	1.06
ZFP36	ZFP36 ring finger protein(ZFP36)	Inconsistent	0.76

*The software predicted activation/inhibition Z-scores of 2.428 (p-value of 5.27 x 10^−14^) and 2.647 (p-value of 7.48 x 10^−6^) for R. conorii- and R. montanensis-infected THP-1 macrophages, respectively. In IPA, Z-scores are used to predict activation or inhibition, where a Z-score ≥ 2.0 represents a statistical significant result of activation while a Z-score ≤ 2.0 yields a prediction of inhibition. Individual genes are color coded according to their contribution for the overall prediction: orange-leads to activation; yellow-findings are inconsistent with the state of downstream function; black-effect is not predicted*.

## Discussion

The ability of many microbial and viral pathogens to modulate host transcriptional responses is a central aspect for pathogenesis (Tran Van Nhieu and Arbibe, 2009; Ashida and Sasakawa, 2014; Lateef et al., 2017). Consequently, the study of host transcriptomic alterations promoted during infection is a useful source of information to understand how pathogens can establish a successful niche inside host cells (Cloney, 2016). The employment of high-throughput sequencing-based transcriptomic technologies has endorsed significant advances in unraveling host-pathogen interactions that contribute for cellular tropism and pathogenicity (Westermann et al., 2012, 2017; Saliba et al., 2017). We have previously shown that *R. conorii* and *R. montanensis*, two SFG *Rickettsia* with different degrees of pathogenicity to humans, display opposite intracellular fates in THP-1 macrophages (Curto et al., 2016). To further understand this phenotypic difference, we herein characterized the early changes in host gene expression in these cells upon challenge with the two SFG *Rickettsia*. This experimental design allowed us to determine not only the host transcriptomic responses induced to clear infection by an avirulent *Rickettsia* but also *R. conorii*-specific alterations that are initiated at a very early time point (1 h) post-infection in THP-1 cells.

Our results revealed that infection with the pathogenic species was able to specifically promote a robust set of alterations in host gene expression. Remarkably, of the significantly DE genes at 1 h post-infection, only 61 genes were found to be common to both infection conditions, whereas 409 genes were specifically altered in *R. conorii*-infected cells and only 25 genes were specifically altered upon challenge with *R. montanensis*. These results indicate that different SFG *Rickettsia*, with distinct pathogenicity attributes, promoted different transcriptional responses in THP-1 macrophages, which ultimately culminate in entirely distinct intracellular fates in the host cell.

We demonstrated that THP-1 cells responded to either *R. conorii* or *R. montanensis* stimuli by augmenting the expression of pro-inflammatory cytokines and chemokines to tackle the infection. However, differences in expression were observed for several other inflammatory-related genes, anticipating a differential host response to each rickettsial species. The observed lower abundance of the CD14 transcript in *R. conorii*-dataset only is one of the examples. Physical interaction between CD14 and TLR4 has been reported, and it is now assumed that a ternary complex incorporating CD14, MD-2, and TLR4 serves to activate LPS signaling (Poltorak et al., 2000; Beutler, 2004). The lower abundance of CD14 mRNA observed upon infection by *R. conorii* might, therefore, affect LPS signaling and downstream pathways. Indeed, reduced levels of CD14 upon infection by *Porhymonas gingivalis* and *Pseudomonas aeruginosa* have been reported as being related with hyporesponsiveness to bacterial challenge (Wilensky et al., 2015; Van Belleghem et al., 2017). Another interesting difference was the *R. conorii*-specific accumulation of transcripts for several genes mapped to NF-KB signaling that have been involved in cell survival, including the gene coding for the growth arrest and DNA damage-inducible 45 protein (GADD45). This protein plays essential roles in connecting NF-κB signaling to MAPK, and it can regulate several cell activities as growth arrest, differentiation, cell survival and apoptosis (Yang et al., 2009). Moreover, differential expression of several genes related to the Jak-STAT signaling pathway was observed in *R. conorii*-infected cells only, further suggesting a specific modulation of immune responses by the pathogenic bacteria. One of these genes is SOCS3 (suppressor of cytokine signaling 3), a cytokine-induced inhibitor that suppresses cytokine receptor-mediated Stat signaling via a negative feedback loop (Mahony et al., 2016). Indeed, high expression of SOCS3 upon infection is well-reported for several bacterial and viral pathogens and it has been linked to pathogenic immune evasion (Yokota et al., 2004, 2005; Narayana and Balaji, 2008; St. John and Abraham, 2009). Our results also showed an accumulation of OSM and MCL1 transcripts. It is reported that the OSM stimulates the expression of MCL1 via JAK1/2-STAT1/3 and CREB and it contributes to bioenergetics improvements and protection against mitochondrial dysfunction (Chang et al., 2015). Upregulation of MCL1 during *Leishmania donovani* infection has been documented as being essential for disease progression by preventing BAK-mediated mitochondria-dependent apoptosis (Giri et al., 2016).

Overall, the first striking observation was the balance between pro- and anti-inflammatory mediators induced upon infection with the pathogenic *Rickettsia*. Modulation of immune signals in the host has been described as a sophisticated strategy developed by successful pathogens to subvert host responses, switching the immune responses into a hyporesponsive state (Gogos et al., 2000). The observed differential expression of genes associated with different signaling transduction pathways (such as TLR, TNFR, NF-κB, or the Jak-STAT pathway), and with other mechanisms of the earliest line of defense against pathogens (e.g., antimicrobial enzymes) in *R. conorii*-infected cells, suggests that this pathogen may be able to modulate innate immune system components at various levels, anticipating the use of complex modulatory mechanisms very early in infection to evade and subvert host responses. This was further substantiated by the observed lack of response to LPS stimulation in *R. conorii*-infected cells, which anticipates a strong modulation of inflammatory as well as macrophage activation responses. An example of manipulation of immune responses by a Gram-negative pathogen is the ability of *Shigella flexneri* to inhibit NF-κB signaling pathways by its Type III effector (OspI), which greatly reduces the acute inflammatory response in macrophages during invasion, as well as the ability of these cells to undergo apoptosis and communicate with other immune cells (Reddick and Alto, 2014). Members of the genus *Rickettsia* do not possess genes to encode a functional Type III secretion system (Gillespie et al., 2015) and therefore must employ other strategies to manipulate the infected host cell. How *R. conorii* can specifically induce this program in infected cells is unclear and currently under investigation.

Another strategy that intracellular pathogens have developed to establish a niche of infection is the ability to control host cell apoptosis to their advantage (Friedrich et al., 2017). Our results revealed that *R. conorii* promoted an increased abundance of several transcripts whose products have been implicated in pro-survival pathways. Some of these gene products (e.g., Bcl-2 protein family members) are targeted by several pathogens to modulate host apoptotic signaling to their advantage (Friedrich et al., 2017). It has been reported that survival of *Mycobacterium tuberculosis* in host macrophages involves resistance to apoptosis by upregulating Bcl-2 and the Bcl-2 like protein Mcl-1 (Sly et al., 2003; Wang et al., 2014); and other studies have also demonstrated that Bcl-2 family members are essential for the survival of *Legionella* by preventing macrophage apoptosis (Speir et al., 2016). Therefore, increased abundance of two Bcl-2 family members (MCL1 and BCL2A1) at an early time post-infection in THP-1 macrophages by *R. conorii* may be a strategy to promote host cell survival and retain a replicative niche. Increased abundance of PIM3 transcripts, a proto-oncogene with serine/threonine kinase activity that can prevent apoptosis, promote cell survival and protein translation, was also observed in *R. conorii*-infected cells only. Interestingly, PIM3 may contribute to tumorigenesis through the delivery of survival signaling through phosphorylation of BAD, which induces the release of the anti-apoptotic protein Bcl-X_L_ (Narlik-Grassow et al., 2014). Another interesting difference was the increased accumulation of SOD2 mRNA observed only in *R. conorii*-infected cells. SOD2 is involved in protection against oxidative stress and, as a result, may have a protective role against cell death (Drane et al., 2001). Modulation of NF-κB signaling pathways has been already described as a strategy developed by *R. rickettsii* to modulate apoptosis over the course of infection in epithelial/endothelial cells (Clifton et al., 1998; Joshi et al., 2003, 2004). We also found no significant effect on PARP-1 cleavage in THP-1 cells at 24 h post-infection, in agreement with a previous study which reported a similar effect for endothelial cells infected with the related pathogen, *R. rickettsii*, for up to 18 h post-infection (Joshi et al., 2003). However, combining our observations at longer time points post-infection, our results point toward a well-designed ability of *R. conorii* to not only manipulate but also to sustain host cell survival early during the infection, suggesting again a complex interference with apoptotic cascades. In contrast, host cell integrity is severely disrupted in *R. montanensis*-infected THP-1 macrophages. Therefore, control of host survival appears to be another key feature exploited by *R. conorii* during THP-1 macrophage infection, and a critical distinguishing factor between these two rickettsial species.

Survival of intracellular pathogens in host cell niches depends on multiple alterations in host cell function, and these changes reflect, in part, the ability of the microbe to alter host cell gene expression (Asrat et al., 2015). Our findings suggest that the drastic difference in the intracellular fate of these two rickettsial species in macrophage-like cells could be the result of the differential ability of *Rickettsia* species to interfere with the regulation of gene expression programs. Indeed, the pathogenic *R. conorii* appears to interfere with a myriad of cellular processes not only to control immediate host responses but apparently promoting changes in several transcriptional and posttranscriptional regulatory elements that may extensively impact host cell functions later in infection. One of these examples is the observed alterations of non-coding RNAs, which have been emerging as key regulatory molecules in controlling gene expression (Duval et al., 2017). Several intracellular bacterial pathogens, such as *Helicobacter pylori, Salmonella* spp., *Mycobacterium tuberculosis*, and many others are able to manipulate the expression profiles of these regulatory molecules resulting in more favorable environmental and physiological conditions for pathogen survival (Das et al., 2016; Duval et al., 2017; Zur Bruegge et al., 2017). Regarding the miRNAs identified in this work, and to our knowledge, only miR-223 has been previously associated with responses to bacterial infection (Staedel and Darfeuille, 2013), suggesting a new role for miR-137, miRNA-424, and miR-663A in these processes. Since this study was not specifically directed to the identification of small non-coding RNAs, we cannot exclude that other miRNAs (as well as other classes of non-coding RNA) may be differentially altered in abundance in this cell type upon infection with *Rickettsia*. The robust and specific changes in different snoRNAs only observed in *R. conorii*-infected cells also raise interesting questions on the role of this class of RNAs for rickettsial survival in THP-1 macrophages. Indeed, it has been reported that snoRNAs can act as mediators of host antiviral response and the activity of regulatory RNAs can be used by viruses to evade innate immunity (Peng et al., 2011; Saxena et al., 2013; Stepanov et al., 2015). We also observed stronger alterations in the abundance of 7SL RNAs in *R. conorii* infected cells. To our knowledge, the relevance of 7SL RNAs in host-pathogen interactions is still mostly unknown and only previously shown during *Leishmania* infection in macrophages (Abell et al., 2004; Misra et al., 2005). Although further studies are required to understand the functional impact of the specific alterations of different ncRNAs by *R. conorii* at 1 h post-infection, our results suggest that this pathogenic bacterium may also exploit these regulatory molecules as a strategy to bolster more favorable conditions for proliferation in macrophages.

Along with ncRNAs, the *R. conorii*-specific alterations in expression of a high number genes associated with RNA polymerase II-dependent host gene expression was another obvious observation. Targeting of Pol II-dependent transcription by pathogens has just been recently reported in urinary tract infections as playing a role in evasion of immune activation (Lutay et al., 2013; Ambite et al., 2016) but, to our knowledge, these mechanisms of pathogen-induced transcriptional modulation are still poorly understood. Of particular importance was the observed DE of several transcription factors (both activators and repressors), which can affect the expression of several other genes and may drastically impact host expression profiles at later stages of infection. Changes in expression of several transcription factors early in infection by *Salmonella typhimurium* were reported to result in unique features of the late transcriptional responses that are required for bacterial intracellular replication (Hannemann et al., 2013). Therefore, our results provide a new example of a pathogenic bacterium capable of inducing a broad effect on RNA Pol II-dependent transcription that deserves to be further studied. Furthermore, our results demonstrate that infection of THP-1 macrophages with *R. conorii* resulted in the increased abundance of transcripts of different members of the AP-1 complex such as FOS, JUN, and JUNB. It is known that the contribution of the AP-1 complex to determination of cell fates critically depends on the relative abundance of AP-1 subunits, the composition of AP-1 dimers, the quality of stimulus, the cell type as well as the cellular environment (Ameyar et al., 2003; Hess et al., 2004). Heterodimers formed by FOS:JUN are more stable complexes with stronger DNA binding affinity when compared to JUN:JUN homodimers, which can further define the host gene expression profiles generated by the AP-1 complex (Halazonetis et al., 1988). Therefore, the observed differential expression of AP-1 subunits may also affect transcriptional programs triggered by each bacterial species. A significant manipulation of the AP-1 transcription factor by *Ebolavirus* (EBOV) infection and its role in host gene expression profiles defining EBOV pathogenesis has been documented (Wynne et al., 2017), urging for the future evaluation of the role of AP1 in *R. conorii* pathogenesis.

Herein, we provide evidence that the gene expression machinery in the host nucleus appears to be a key target of *R. conorii* interference, likely contributing to modulate host processes to establish a favorable cell environment for bacterial survival and proliferation in THP-1 macrophages. Therefore, one of the most important questions that now emerge is how *R. conorii* is regulating the response of the genome. Several strategies have been identified for other pathogenic bacteria, and the identification of microbial effectors that directly target and alter host gene expression programs at the level of transcriptional regulation has been emerging as a new field of research (Bierne and Cossart, 2012; Reddick and Alto, 2014; Asrat et al., 2015). In SFG *Rickettsia*, the nature and function of bacterial effectors are still mostly elusive. However, in other intracellular pathogenic bacteria of the related genera *Ehrlichia* and *Anaplasma*, recent studies identified ankyrin repeat-containing proteins (Anks) as key virulence factors by their ability to affect host gene expression profiles (Pan et al., 2008; Dumler et al., 2016). Of note, the Ank gene homolog *Rickettsia* Ankyrin Repeat Protein 2 (RARP-2) is present in *R. conorii* genome but absent in *R. montanensis*, which might, in part, explain the differential expression programs generated upon infection (Gillespie et al., 2015). Therefore, further studies exploring the potential role of RARP-2 as a virulence factor in rickettsial species should be promptly addressed.

By unraveling early alterations in host gene expression profiles upon infection of macrophage-like cells with two SFG rickettsial species with different pathogenicity attributes, this work contributes new insights on how host cell functions and multiple signaling events respond to either clear an infection or to be exploited to the own benefit of a pathogen. Combined, these findings raise the exciting hypothesis that manipulation of host gene expression programs may be a virulence strategy deployed by pathogenic rickettsiae to proliferate in macrophage-like cells. These results will help to guide future research with valuable resources that can be used to expand our understanding of the complex network of host-rickettsiae interactions, including deciphering the nature and function of rickettsial virulence effectors as well as the role of phagocytic cells in the pathogenesis of rickettsial diseases.

## Author Contributions

PC, IS, and JM conceptualized the study. PC, SR, and IS conducted the investigation. PC and SR conducted the formal analysis. PC and IS wrote the original draft of the manuscript. PC, IS, and JM wrote, reviewed and edited the manuscript. IS and JM supervised the study. JM provided resources. IS and JM acquired the funding.

### Conflict of Interest Statement

The authors declare that the research was conducted in the absence of any commercial or financial relationships that could be construed as a potential conflict of interest.
