# Supplementary material for: Macrophages Infected by a Pathogen and a Non-pathogen Spotted Fever Group Rickettsia Reveal Differential Reprogramming Signatures Early in Infection
Source: Front Cell Infect Microbiol. 2019 Apr 10;9:97. doi: 10.3389/fcimb.2019.00097 (PMC6467950; doi:10.3389/fcimb.2019.00097)
Supplement: Supplementary file 3 [file Table_3.DOCX]

**Supplementary Table 3**. Genes targeted for qRT-PCR validation, primer sequences, and calculated log_2_ fold changes by qRT-PCR and RNA-seq in each respective experimental condition (Associated with Figure 1).

|  | **Gene**  **I.D.** | **Primer Name** | **Primer Sequence** | **Product Size**  **(b. p.)** | **Log_2_Fold Change (*R.con*/Uninf.)** | | **Log_2_Fold**  **Change (*R.mont*/Uninf.)** | |
| --- | --- | --- | --- | --- | --- | --- | --- | --- |
|  |  |  |  |  | **qRT-PCR** | **RNA-seq** | **qRT-PCR** | **RNA-seq** |
| 1 | B2M | B2M_F | GTG CTC GCG CTA CTC TCT C | 50 | 1.2 | 0.1 | 0.3 | 0.1 |
|  |  | B2M_R | GGA CTA CGC TGG ATA GCC TC |  |  |  |  |  |
| 2 | BTG2 | BTG2_F | TGA GGT GTC CTA CCG CAT TG | 56 | 3.2 | 3.0 | 1.1 | 1.1 |
|  |  | BTG2_R | CCT CCT CGT ACA AGA CGC AG |  |  |  |  |  |
| 3 | CD69 | CD69_F | AGG AAC ACT GGG TTG GAC TG | 50 | 4.2 | 3.7 | 1.5 | 2.2 |
|  |  | CD69_R | CCA CTT CCA TGG GTG ACC AG |  |  |  |  |  |
| 4 | EGR1 | EGR1_F | AAG TTT GCC AGG AGC GAT GA | 65 | 3.0 | 3.3 | 0.8 | 0.8 |
|  |  | EGR1_R | TTC TTG TCC TTC TGC CGC AA |  |  |  |  |  |
| 5 | EMC7 | EMC7_F | TCT GGC AAA TCT AGC AGC GG | 55 | 0.3 | -0.2 | 1.1 | 0.1 |
|  |  | EMC7_R | TTT TGC CAG CCC CAC TTT TG |  |  |  |  |  |
| 6 | G6PD | G6PD_F | TTT GCC CGC AAC TCC TAT GT | 79 | 0.0 | -0.1 | 0.0 | 0.0 |
|  |  | G6PD_R | GGG CAT TCA TGT GGC TGT TG |  |  |  |  |  |
| 7 | IER3 | IER3_F | CTT CGG AGC CCT CGG ACT A | 52 | 3.9 | 3.1 | 2.2 | 1.8 |
|  |  | IER3_R | TGT TGC TGG AGG AAA GTG CT |  |  |  |  |  |
| 8 | KLF10 | KLF10_F | AAG GCG CTG TCA TGT TTG TG | 58 | 3.3 | 2.4 | 0.7 | 0.9 |
|  |  | KLF10_R | ACC GGA GGC TTT GAA CTC TG |  |  |  |  |  |
| 9 | MTRNR2L6 | MTRNR2L6_F | CAC GAG GGT TCA GCT GTC TC | 59 | 0.4 | 1.0 | 0.6 | 1.9 |
|  |  | MTRNR2L6_R | CCT CTT CAC AGG CAG GTC AG |  |  |  |  |  |
| 10 | OTUD1 | OTUD1_F | CCG ACC ATC TCG ACC ACT TC | 70 | 3.2 | 2.5 | 1.1 | 1.1 |
|  |  | OTUD1_R | TGG GCA GCA GCG ATG ATA AA |  |  |  |  |  |
| 11 | PP1R15A | PP1R15A_F | GGC ATG TAT GGT GAG CGA GA | 59 | 2.3 | 2.7 | 0.6 | 0.8 |
|  |  | PP1R15A_R | GCA AAT TGA CTT CCC TGC CC |  |  |  |  |  |
