# Supplementary material for: Macrophages Infected by a Pathogen and a Non-pathogen Spotted Fever Group Rickettsia Reveal Differential Reprogramming Signatures Early in Infection
Source: Front Cell Infect Microbiol. 2019 Apr 10;9:97. doi: 10.3389/fcimb.2019.00097 (PMC6467950; doi:10.3389/fcimb.2019.00097)
Supplement: Supplementary file 5 [file Table_5.DOCX]

**Supplementary Table 5.** DE genes with changed abundance specifically upon infection of THP-1 macrophages with *R. montanensis* are categorized using DAVID Bioinformatic Resources 6.8. DE genes are categorized according to GO terms: biological process and cellular component, and KEGG canonical pathways.

| ***R. montanensis*-specific DE genes (25 genes)** | **GO Biological Process** | **Number of genes** | **% Gene count** | **p-value** |
| --- | --- | --- | --- | --- |
|  | Regulation of G-protein coupled receptor protein signaling pathway | 3 | 12 | 1.8 x 10^-2^ |
|  | Positive regulation of transcription, DNA-templated | 3 | 12 | 2.3 x 10^-2^ |
|  | Positive regulation of transcription from RNA polymerase II promoter | 2 | 8 | 7.5 x 10^-2^ |
|  | **GO Cellular Component** | **Number of genes** | **% Gene count** | **p-value** |
|  | Nuclear speck | 2 | 8 | 8.5 x 10^-2^ |
