# Supplementary material for: Macrophages Infected by a Pathogen and a Non-pathogen Spotted Fever Group Rickettsia Reveal Differential Reprogramming Signatures Early in Infection
Source: Front Cell Infect Microbiol. 2019 Apr 10;9:97. doi: 10.3389/fcimb.2019.00097 (PMC6467950; doi:10.3389/fcimb.2019.00097)
Supplement: Supplementary file 7 [file Table_7.DOCX]

**Supplementary Table 7**. Selected genes involved in innate immune responses targeted for qRT-PCR validation, primer sequences, and calculated log_2_ fold changes by qRT-PCR and RNA-seq in *R. conorii*-infected THP-1 macrophages (Associated with Figure 3, Figure S2).

|  | **Gene**  **I.D.** | **Primer Name** | **Primer Sequence** | **Log_2_Fold Change (*R.con*/Uninf.)** | |
| --- | --- | --- | --- | --- | --- |
|  |  |  |  | **qRT-PCR** | **RNA-seq** |
| 1 | BCL3 | BCL3_F | CAA GAA CTG CCA CAA CGA CA | 1.5 | 1.9 |
|  |  | BCL3_R | GAT GTC GAT GAC CCT GCG G |  |  |
| 2 | CCL3 | CCL3_F | TGT CAT CTT CCT AAC CAA GAG AGG | 6.5 | 5.6 |
|  |  | CCL3_R | TAT TTC TGG ACC CAC TCC TCA |  |  |
| 3 | CCL4L2 | CCL4L2_F | GGA AGG ATC CCA TCC ACC AG | 11.4 | 8.6 |
|  |  | CCL4L2_R | GGT AGG CAT CTT CCT CTG CC |  |  |
| 4 | CXCL3 | CXCL3_F | AGA AAG CTT GTC TCA ACC CCG | 7.8 | 5.6 |
|  |  | CXCL3_R | GGT GCT CCC CTT GTT CAG TA |  |  |
| 5 | ICAM1 | ICAM1_F | GGT AGC AGC CGC AGT CAT AA | 1.9 | 1.6 |
|  |  | ICAM1_R | TCC CTT TTT GGG CCT GTT GT |  |  |
| 6 | IL1A | IL1A_F | CTT CTG GGA AAC TCA CGG CA | 0.7 | 5.6 |
|  |  | IL1A_R | AGC ACA CCC AGT AGT CTT GC |  |  |
| 7 | NFKBIA | NFKBIA_F | TGT GCT TCG AGT GAC TGA CC | 4.7 | 5.3 |
|  |  | NFKBIA_R | TCA CCC CAC ATC ACT GAA CG |  |  |
| 8 | PTGS2 | PTGS2_F | GGC CAT GGG GTG GAC TTA AA | 4.4 | 4.9 |
|  |  | PTGS2_R | TGA AAA GGC GCA GTT TAC GC |  |  |
