# Supplementary material for: Macrophages Infected by a Pathogen and a Non-pathogen Spotted Fever Group Rickettsia Reveal Differential Reprogramming Signatures Early in Infection
Source: Front Cell Infect Microbiol. 2019 Apr 10;9:97. doi: 10.3389/fcimb.2019.00097 (PMC6467950; doi:10.3389/fcimb.2019.00097)
Supplement: Supplementary file 8 [file Table_8.DOCX]

**Supplementary Table 8.** Fold change of DE genes categorized in JAK/STAT pathway according to KEGG pathways in THP-1 cells infected with *R. conorii* or *R. montanensis*.

| **Gene I.D.** | **Gene description** | **Log_2_ Fold Change (*R.con*/Uninf.)** | **Log_2_ Fold Change (*R.mont*./Uninf.)** | **Biological**  **Function** |
| --- | --- | --- | --- | --- |
| IL23A | Interleukin 23 subunit alpha | 1.38 | n.s. | Cytokine-Cytokine receptor interaction |
| OSM | Oncostatin M | 3.99 | n.s |  |
| SOCS3 | Suppressor of cytokine signaling 3 | 4.89 | n.s | Negative feedback loop |
| MCL1 | Induced myeloid leukemia cell differentiation protein | 0.78 | n.s | Anti-Apoptosis |
| MYC | v-myc avian myelocytomatosis viral oncogene homolog | 0.71 | n.s. | Cell-cycle |
| CDKN1A | Cyclin dependent kinase inhibitor 1A | 0.67 | n.s. |  |

n.s. – Fold change of the specific gene is not statistically significant upon infection.
