# Supplementary material for: Macrophages Infected by a Pathogen and a Non-pathogen Spotted Fever Group Rickettsia Reveal Differential Reprogramming Signatures Early in Infection
Source: Front Cell Infect Microbiol. 2019 Apr 10;9:97. doi: 10.3389/fcimb.2019.00097 (PMC6467950; doi:10.3389/fcimb.2019.00097)
Supplement: Supplementary file 9 [file Table_9.DOCX]

**Supplementary Table 9.** Contribution of DE genes in *R. conorii*- and *R. montanensis*-infected THP-1 macrophages for negative regulation of apoptotic process according to biological process GO terms (GO:0043066).

| **Gene I.D.** | **Gene description** | **Log_2_ Fold Change (R.con/Uninf.)** | **Log_2_ Fold Change (R.mont./Uninf.)** |
| --- | --- | --- | --- |
| BCL2A1 | BCL2 related protein A1 | 1.2 | n.s. |
| BCL3 | B-cell CLL/lymphoma 3 | 1.87 | n.s |
| CDKN1A | cyclin dependent kinase inhibitor 1A | 0.67 | n.s. |
| CITED2 | Cbp/p300 interacting transactivator with Glu/Asp rich carboxy-terminal domain 2 | 0.74 | n.s |
| DUSP1 | Dual specificity phosphatase 1 | 4.68 | 2.37 |
| ID1 | Inhibitor of DNA binding 1, HLH protein | -1.23 | n.s. |
| IER3 | Immediate early response 3 | 3.09 | 1.76 |
| MCL1 | BCL2 family apoptosis regulator | 0.78 | n.s. |
| MYC | v-myc avian myelocytomatosis viral oncogene homolog | 0.71 | n.s. |
| NFKBIA | NFKB inhibitor alpha | 5.29 | 3.16 |
| NUAK2 | NUAK family kinase 2 | -0.75 | n.s |
| PHB2 | Prohibitin 2 | -0.67 | n.s. |
| PIM3 | Pim-3 proto-oncogene, serine/threonine kinase | 2.06 | n.s. |
| PLK3 | Polo like kinase 3 | 1.14 | n.s. |
| RIPK2 | Receptor interacting serine/threonine kinase 2 | 1.32 | n.s. |
| SOCS3 | Suppressor of cytokine signaling 3 | 4.89 | n.s. |
| SOD2 | Superoxide dismutase 2, mitochondrial | 3.25 | n.s. |
| TIMP1 | TIMP metallopeptidase inhibitor 1 | -0.61 | n.s. |
| TNFAIP8 | TNF alpha induced protein 8 | 0.86 | n.s. |
