# Supplementary material for: Macrophages Infected by a Pathogen and a Non-pathogen Spotted Fever Group Rickettsia Reveal Differential Reprogramming Signatures Early in Infection
Source: Front Cell Infect Microbiol. 2019 Apr 10;9:97. doi: 10.3389/fcimb.2019.00097 (PMC6467950; doi:10.3389/fcimb.2019.00097)
Supplement: Supplementary file 10 [file Table_10.DOCX]

**Supplementary Table 10.** Fold change of non-coding transcripts in *R. conorii*- or *R. montanensis*-infected THP-1 macrophages (Associated with Figure 8).

|  | **Gene I.D.** | **Log_2_ Fold Change (*R.con*/Uninf.)** | **Log_2_ Fold Change (*R.mont*./Uninf.)** |
| --- | --- | --- | --- |
| **Long intergenic non-protein coding RNA** | LINC00235 | -0.68 | n.s. |
|  | LINC00346 | 2.93 | n.s. |
|  | LINC00599 | -1.77 | n.s. |
|  | LINC00638 | -1.35 | n.s. |
|  | LINC00896 | -1.75 | n.s. |
|  | LINC00899 | -1.23 | n.s. |
|  | LINC01125 | -0.62 | n.s. |
|  | LINC01230 | -1.13 | n.s. |
|  | LINC01353 | 2.56 | n.s. |
|  | LINC01686 | n.s. | 0.91 |
|  | LINC02202 | 1.89 | n.s. |
| **Micro RNAs** | MIR137 | 2.13 | n.s. |
|  | MIR223 | -0.89 | n.s. |
|  | MIR424 | -0.97 | n.s. |
|  | MIR663A | n.s. | 1.2 |
| **7SK small nuclear RNAs** | RN7SKP1 | -0.92 | -1.05 |
|  | RN7SKP48 | -1.57 | n.s. |
|  | RN7SKP91 | -0.56 | n.s. |
|  | RN7SKP172 | -1.76 | n.s. |
|  | RN7SKP286 | -0.90 | n.s. |
| **7SL cytoplasmic RNAs** | RN7SL18P | 1.10 | n.s. |
|  | RN7SL33P | 1.74 | 1.32 |
|  | RN7SL40P | 0.66 | n.s. |
|  | RN7SL44P | 2.54 | 1.92 |
|  | RN7SL87P | 1.23 | n.s. |
|  | RN7SL97P | 0.95 | n.s. |
|  | RN7SL140P | 16.49 | n.s. |
|  | RN7SL148P | 1.29 | n.s. |
|  | RN7SL162P | 1.47 | n.s. |
|  | RN7SL184P | 0.91 | n.s. |
|  | RN7SL215P | 15.85 | n.s. |
|  | RN7SL338P | 1.60 | n.s. |
|  | RN7SL357P | 0.91 | n.s. |
|  | RN7SL471P | 1.30 | n.s. |
|  | RN7SL472P | 0.82 | n.s. |
|  | RN7SL478P | 1.19 | 1.16 |
|  | RN7SL513P | 1.29 | n.s. |
|  | RN7SL521P | 1.51 | n.s. |
|  | RN7SL555P | 0.93 | n.s. |
|  | RN7SL658P | 1.83 | n.s. |
|  | RN7SL679P | 1.18 | n.s. |
|  | RN7SL767P | 0.55 | n.s. |
|  | RN7SL824P | 1.35 | 0.76 |
|  | RN7SL836P | 1.78 | n.s. |
|  | RN7SL838P | 1.38 | n.s. |
| **5S ribosomal RNAs** | RNA5-8SP2 | -19.29 | n.s. |
|  | RNA5-8SP6 | -2.95 | -1.68 |
|  | RNA5SP202 | -3.19 | n.s. |
| **U-RNAs** | RNU1-7P | n.s. | 1.18 |
|  | RNU1-17P | n.s. | 1.24 |
|  | RNU1-18P | n.s. | 0.92 |
|  | RNU1-32P | 3.19 | n.s. |
|  | RNU1-55P | n.s. | 1.48 |
|  | RNU1-89P | -0.83 | n.s. |
|  | RNU1-148P | -1.03 | 1.18 |
|  | RNU2-14P | n.s. | 1.03 |
|  | RNU2-8P | n.s. | 1.31 |
|  | RNU4-1 | -1.05 | n.s. |
|  | RNU4-2 | -0.89 | n.s. |
|  | RNU5A-1 | -0.95 | 0.95 |
|  | RNU5A-2P | n.s. | 1.67 |
|  | RNU5B-1 | -0.77 | n.s. |
|  | RNU5D-1 | -0.66 | 0.71 |
|  | RNU5E-1 | -0.76 | n.s. |
|  | RNU5F-1 | -0.95 | n.s. |
|  | RNU6ATAC | -0.60 | n.s. |
| **snoRNAs** | SNORA23 | -0.98 | n.s. |
|  | SNORA2A | -0.83 | n.s. |
|  | SNORA37 | -1.03 | n.s. |
|  | SNORA38B | -0.7 | n.s. |
|  | SNORA47 | -0.84 | n.s. |
|  | SNORA54 | -0.97 | n.s. |
|  | SNORA5C | -1.33 | n.s. |
|  | SNORA71B | -0.84 | n.s. |
|  | SNORA74B | -0.98 | n.s. |
|  | SNORA79 | -0.89 | n.s. |
|  | SNORA7B | -0.87 | n.s. |
|  | SNORA80B | -0.85 | n.s. |
|  | SNORA80E | -1 | n.s. |
|  | SNORD17 | -0.85 | n.s. |
|  | SNORD3C | -0.69 | n.s. |
|  | SNORD60 | -0.8 | n.s. |
|  | SNORD67 | -0.74 | n.s. |
|  | SNORD92 | -0.69 | n.s. |
|  | SCARNA1 | -1.16 | n.s. |
|  | SCARNA8 | -0.97 | n.s. |
|  | SCARNA13 | -0.55 | n.s. |
|  | FAM153C | 1.56 | n.s. |
|  | FAM71A | 3.23 | n.s. |
|  | FAM83C-AS1 | -15.29 | n.s. |
|  | FAM13A-AS1 | n.s. | 2.59 |

n.s. – Fold change of the specific gene is not statistically significant upon infection.
