# Supplementary material for: Macrophages Infected by a Pathogen and a Non-pathogen Spotted Fever Group Rickettsia Reveal Differential Reprogramming Signatures Early in Infection
Source: Front Cell Infect Microbiol. 2019 Apr 10;9:97. doi: 10.3389/fcimb.2019.00097 (PMC6467950; doi:10.3389/fcimb.2019.00097)
Supplement: Supplementary file 12 [file Data_Sheet_1.PDF]

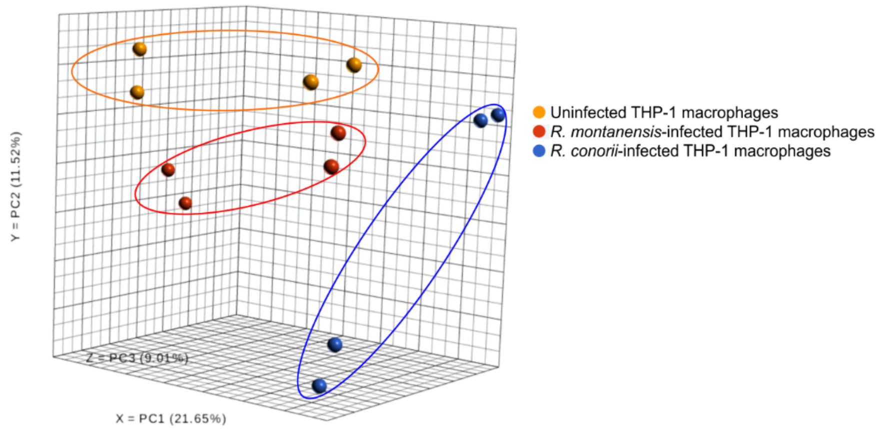

Supplementary Figure 1. 3D principal component analysis (PCA) plots of global transcriptome profiles. 3D PCA plot was performed by importing the mapped read (BAM) files of all RNA-seq data into Partek® Flow® Software. Each sample is represented by a dot and the color label corresponds to the sample group. Orange dots correspond to uninfected THP-1 macrophages, red dots correspond to *R. montanensis*-infected THP-1 macrophages and blue dots correspond to *R. conorii*-infected THP-1 macrophages. The axes show the first three principal components, with the fraction of explained variance in the parenthesis.
