# Supplementary material for: Macrophages Infected by a Pathogen and a Non-pathogen Spotted Fever Group Rickettsia Reveal Differential Reprogramming Signatures Early in Infection
Source: Front Cell Infect Microbiol. 2019 Apr 10;9:97. doi: 10.3389/fcimb.2019.00097 (PMC6467950; doi:10.3389/fcimb.2019.00097)
Supplement: Supplementary file 13 [file Data_Sheet_2.PDF]

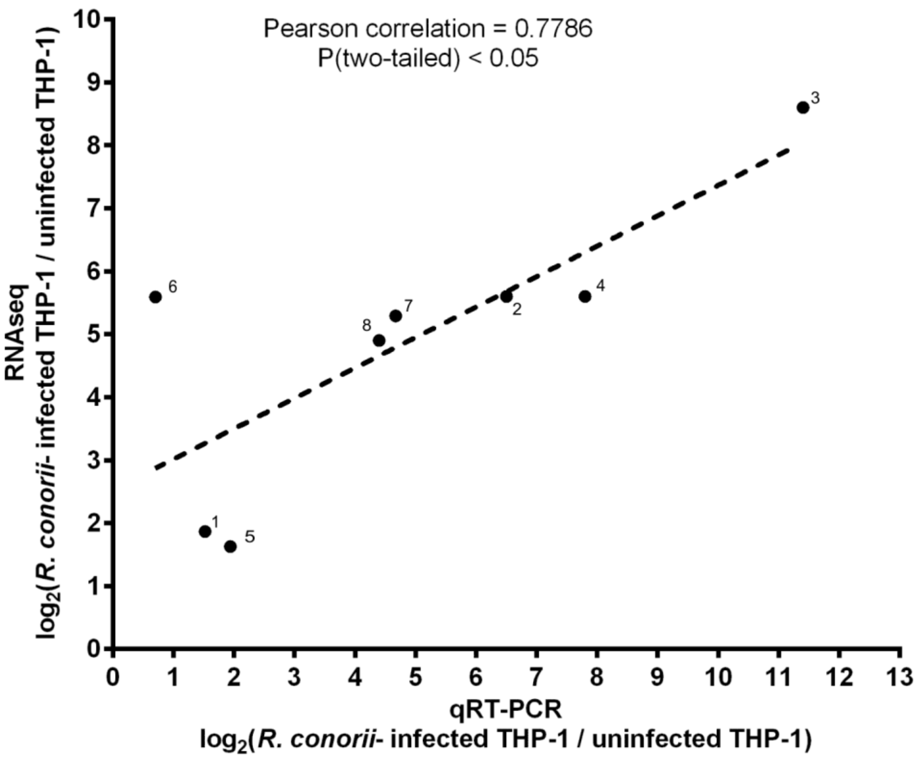


**Supplementary Figure 2. Validation of RNA-seq data of selected genes involved in innate immune responses.** Transcriptional fold changes of 8 genes (Figure 3) were validated by comparing the transcriptional fold changes determined by RNA-seq and an independent method (q-RT-PCR) for *R. conorii*-infected cells. Pearson analysis of correlation and respective significant test (two-tailed) were performed in GraphPad Prism. $r = 0.7786$, $N = 8$, $p < 0.05$. Gene labeling: 1-BCL3; 2-CCL3; 3-CCL4L2; 4-CXCL3; 5-ICAM1; 6-IL1A; 7-NFKBIA; 8-PTGS2; See also Table S7.
